# FGL2 promotes tumour growth and attenuates infiltration of activated immune cells in melanoma and ovarian cancer models

**DOI:** 10.1038/s41598-024-51217-1

**Published:** 2024-01-08

**Authors:** Kristianne J. C. Galpin, Galaxia M. Rodriguez, Vincent Maranda, David P. Cook, Elizabeth Macdonald, Humaira Murshed, Shan Zhao, Curtis W. McCloskey, Andrzej Chruscinski, Gary A. Levy, Michele Ardolino, Barbara C. Vanderhyden

**Affiliations:** 1 Cancer Therapeutics Program, Ottawa Hospital Research Institute, 501 Smyth Road, Ottawa, ON K1H 8L6 Canada; 2 Department of Cellular and Molecular Medicine, University of Ottawa, 451 Smyth Road, Ottawa, ON K1H 8M5 Canada; 3 Multi-Organ Transplant Program, Toronto General Hospital, University Health Network, University of Toronto, Toronto, ON Canada; 4 Department of Biochemistry, Microbiology and Immunology, University of Ottawa, 451 Smyth Road, Ottawa, ON K1H 8M5 Canada

**Keywords:** Cancer immunotherapy, Tumour immunology

## Abstract

The tumour microenvironment is infiltrated by immunosuppressive cells, such as regulatory T cells (Tregs), which contribute to tumour escape and impede immunotherapy outcomes. Soluble fibrinogen-like protein 2 (sFGL2), a Treg effector protein, inhibits immune cell populations, via receptors FcγRIIB and FcγRIII, leading to downregulation of CD86 in antigen presenting cells and limiting T cell activation. Increased FGL2 expression is associated with tumour progression and poor survival in several different cancers, such as glioblastoma multiforme, lung, renal, liver, colorectal, and prostate cancer. Querying scRNA-seq human cancer data shows FGL2 is produced by cells in the tumour microenvironment (TME), particularly monocytes and macrophages as well as T cells and dendritic cells (DCs), while cancer cells have minimal expression of FGL2. We studied the role of FGL2 exclusively produced by cells in the TME, by leveraging *Fgl2* knockout mice. We tested two murine models of cancer in which the role of FGL2 has not been previously studied: epithelial ovarian cancer and melanoma. We show that absence of FGL2 leads to a more activated TME, including activated DCs (CD86+, CD40+) and T cells (CD25+, TIGIT+), as well as demonstrating for the first time that the absence of FGL2 leads to more activated natural killer cells (DNAM-1+, NKG2D+) in the TME. Furthermore, the absence of FGL2 leads to prolonged survival in the B16F10 melanoma model, while the absence of FGL2 synergizes with oncolytic virus to prolong survival in the ID8-*p53*^*−/−*^*Brca2*^*−/−*^ ovarian cancer model. In conclusion, targeting FGL2 is a promising cancer treatment strategy alone and in combination immunotherapies.

## Introduction

Fibrinogen-like protein 2 (FGL2) is a multi-functional protein expressed in macrophages^1,2^, T cells (notably Tregs and TIGIT+ Tregs)^3–6^, natural killer (NK) cells^7^, dendritic cells (DCs)^8^, myeloid derived suppressor cells (MDSCs)^9^, endothelial cells^10,11^, and cancer associated fibroblasts (CAFs)^12^. FGL2 can be found in either a secreted form (sFGL2)^3,5^ with immunosuppressive properties, or a membrane-bound form (mFGL2)^13^ with prothrombinase activity, with both forms derived from the same transcript. sFGL2 is involved in numerous immunoregulatory processes as shown in viral^14,15^ and parasitic infections^16,17^, promoting allo/xenograft tolerance^3,18,19^, and restricting progression of autoimmune diseases such as glomerulonephritis^5^, hepatitis^20^, myocarditis^21^, and arthritis^22^. It also has protective roles in inflammatory conditions such as sepsis^9^ and colitis^23,24^.

sFGL2 acts directly on immune cell populations via its receptors FcγRIIB and FcγRIII^5^, to promote immunosuppression^3,20^. sFGL2 leads to B cell apoptosis^5^, or downregulation of MHC-II and co-stimulatory molecules (CD86, CD83, CD80, and CD40) on DCs and macrophages^3,5,25^. CD25+ FOXP3+ Tregs have high expression of FGL2, suggesting that FGL2 is a Treg effector that suppresses effector T cell proliferation^3,5,26^. FOXP3+ Tregs expressing TIGIT (T cell immunoreceptor with Ig and ITIM domains) upregulates expression of FGL2 upon activation, and selectively suppresses the pro-inflammatory T helper 1 (Th1) and Th17, but not Th2, responses^20,27^. In a subset of Batf3-dependent migratory DCs, sFGL2 blocks GM-CSF induction of CD103 expression, limiting CD8+ T cell activation^28^. In a subset of CD8+ T cells expressing FcγRIIB^20,29^, sFGL2 promotes apoptosis^29^ or suppresses proliferation^20^. Increasing evidence in several disease systems highlights the role of sFGL2 in promoting M2 macrophage polarization^19,24,30,31^. sFGL2 is therefore an immunomodulatory protein which targets multiple immune cell types, promoting immunosuppression.

High FGL2 expression is associated with tumour progression and poor survival in a number of different cancers, such as glioblastoma multiforme^28,30,31^, colorectal^32^, lung^12^, renal^33^, liver^25,34,35^, and prostate cancer^36^. Previously, mFGL2 was shown to accelerate tumour progression by promoting tumorigenesis and angiogenesis in some of these cancers^33–36^. In glioblastoma mouse models, elevated sFGL2 was associated with tumour infiltration of immunosuppressive immune cell populations (Tregs, MDSCs, M2 macrophages) and suppressing CD103+ DC differentiation, leading to tumour progression^28,30,31^. However, in all these studies the assumption was that FGL2 was expressed by the cancer cells and, in mechanistic studies, cancer cells were engineered to overexpress FGL2. In one study using murine models of hepatocellular cancer, researchers investigated the role of FGL2 produced by stromal and immune cells rather than cancer cells in the TME. In the absence of FGL2 in the TME, tumour infiltrating T cells and DCs were more activated^25^. In both the glioma and hepatocellular models, blocking sFGL2 with an antibody immunotherapy significantly prolonged survival^25,30,31^.

Herein, we further examined the expression of FGL2 in different types of tumours, identified its cellular targets, and explored its potential as a therapeutic target. We found that in many types of cancer, FGL2 is produced by immune and stromal cells in the TME, particularly monocytes and macrophages, and to a lesser extent, by T cells and DCs, while cancer cells have minimal FGL2 expression. We therefore studied the role of FGL2 exclusively produced by immune and stromal cells in the TME, in two models of cancer in which the role of FGL2 was not previously studied. Based on our expertise in ovarian cancer we chose to study the role of FGL2 in epithelial ovarian cancer and expanded our study to compare and contrast the role of FGL2 in a common model for immunotherapy research, the B16F10 melanoma model. In this context, the absence of FGL2 lead to increased infiltration of activated DCs and T cells. We also report for the first time, increased presence of activated NK cells. Importantly, absence of FGL2 led to prolonged survival in the B16F10 model of melanoma, and it promoted a stronger response to oncolytic virus immunotherapy to prolong survival in the ID8-*p53*^*−/−*^*Brca2*^*−/−*^ model of ovarian cancer.

## Results

### Cellular expression of FGL2 and its receptor in tumours

**Figure 1:**
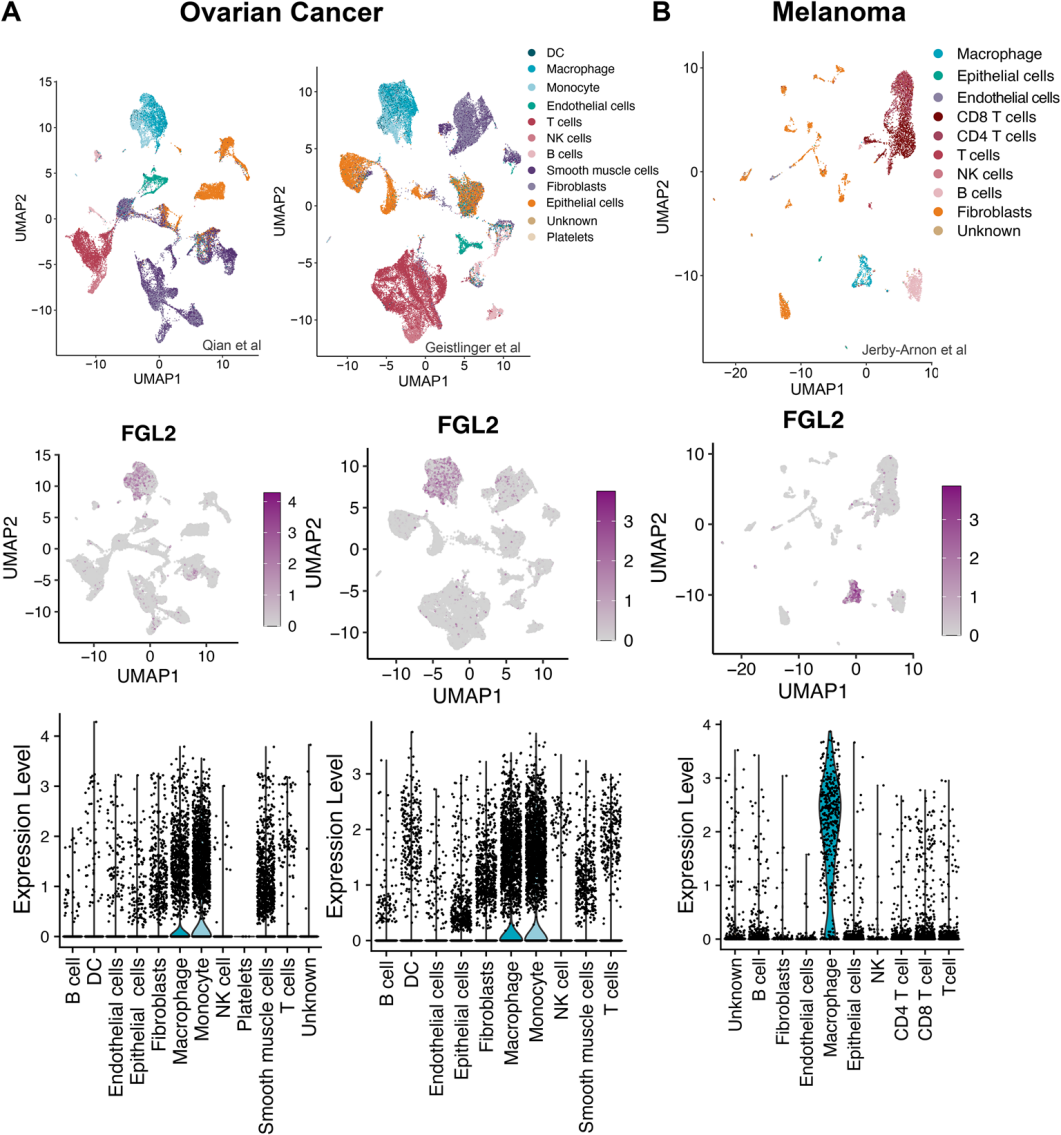
FGL2 is primarily expressed in macrophages of human ovarian cancer and melanoma. (**A**) UMAP embedding of scRNA-seq data from human ovarian cancer datasets [left: Qian et al.^39^ (n = 5), right: Geistlinger et al.^40^ (n = 5)] depicting cell clusters (top) with accompanying heatmap (middle) and violin plot (bottom) displaying the level of FGL2 expression in each cell type cluster. (**B**) UMAP embedding of scRNA-seq data from a human melanoma dataset^38^ (n = 33) depicting cell clusters (top) with accompanying heatmap (middle) and violin plot (bottom) displaying the level of FGL2 expression in each cell type cluster.

To determine the cellular expression of *Fgl2,* we first examined publicly available and previously compiled^37^ scRNA-seq datasets of tumours from patients with melanoma^38^ and ovarian cancer^39,40^. While many previous studies have suggested FGL2 expression by cancer cells may result in a less favourable prognosis^28,30–33^, we found that *Fgl2* was expressed in the macrophage/monocyte and DC populations with minimal expression in the cancer (epithelial) cells (Fig. 1). When analyzing the FGL2 receptors, we found that FcγRIIB was primarily expressed by macrophages/monocytes and DCs, as well as by B cells, and the receptor FcγRIII was primarily expressed in macrophages/monocytes and DCs, as well as NK cells (Fig. S1), suggesting FGL2 has both autocrine and paracrine activity in the TME. Similarly, *Fgl2* expression was observed in macrophages/monocytes and DCs, with minimal cancer cell expression found when analyzing datasets from lung^39,41^, breast, and colorectal^39,43^ tumours (Fig. S2).

To further assess sFGL2 in ovarian cancer, sFGL2 concentrations were quantified in the ascites fluid of ovarian cancer vs. non-cancer (cirrhotic) patients. sFGL2 was abundant in ascites fluid from ovarian cancer patients and at elevated levels when compared to non-cancer ascites (Fig. S3A). A broad assessment of available human cancer cell lines by qPCR shows that FGL2 is not detected in epithelial cancer cell lines, but is detectable in glioblastoma cell lines, monocyte-derived THP-1 cells, and T cell-derived Jurkat cells (Fig. S3B). Importantly, while some previous studies have proposed FGL2 expression in cancer cells, the commonly used antibody from Abnova (clone 6D9) shows a non-specific band in cell lines where FGL2 is not detectable by qPCR **(**Fig. S3C). In contrast, a polyclonal antibody previously generated by Dr. Gary Levy^14^ is specific and recognizes only FGL2 (Fig. S3C). As the results indicate that FGL2 is not expressed in most cancer cells, but is elevated in ovarian cancer ascites fluid, sFGL2 is an immunomodulatory protein of interest in the TME. Therefore, we sought to determine how FGL2 expressed in immune cells in the TME might promote immunosuppression and how it might influence tumour progression and survival.

### Absence of FGL2 slows tumour progression and prolongs survival of B16F10 melanoma models

**Figure 2:**
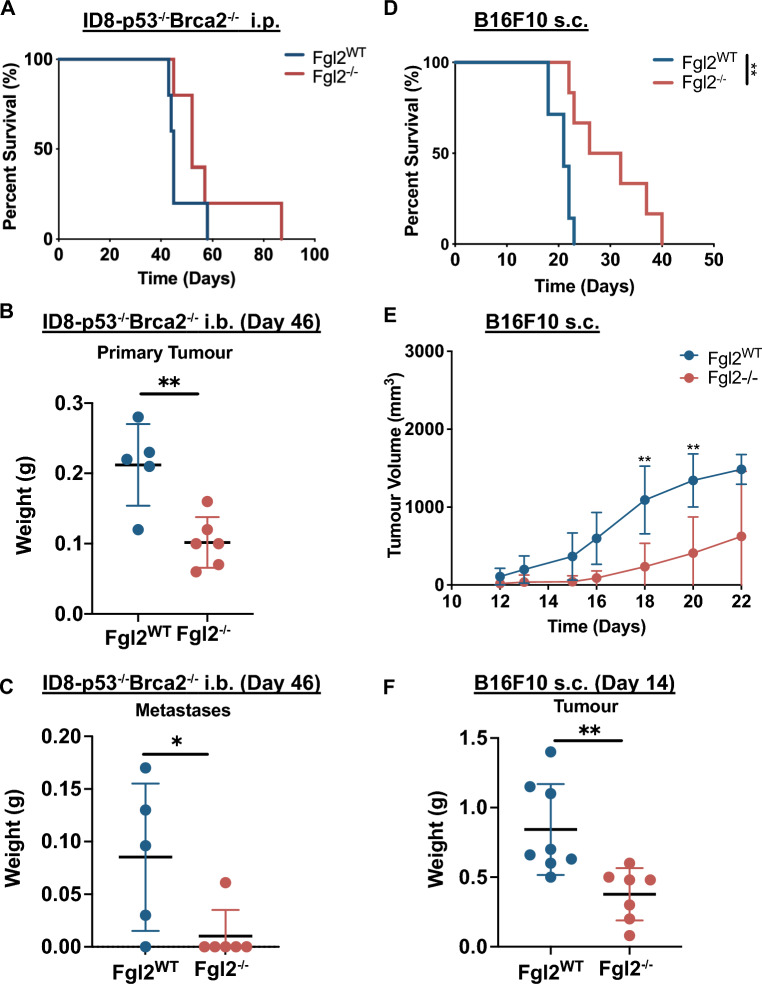
Absence of FGL2 reduces tumour burden in ovarian and melanoma tumour-bearing mice. In the ID8-*p53*^*−/−*^*Brca2*^*−/−*^ ovarian cancer model, *Fgl2*^*WT*^ or *Fgl2*^*−/−*^ mice received **(A)** an i.p. injection of 5 × 10^6^ cells (n = 6/group) on day 0 to assess survival (Log-Rank test, p = 0.2) or bilateral i.b. injections of 0.15 × 10^6^ ID8-*p53*^*−/−*^*Brca2*^*−/−*^ cells (n = 5–6/group) to assess (**B)** primary tumour burden or **(C)** peritoneal metastatic burden on day 46. Significance determined by Student’s t test, *p ≤ 0.05, **p ≤ 0.01. In the B16F10 melanoma model, *Fgl2*^*WT*^ or *Fgl2*^*−/−*^ mice received a s.c. of 2 × 10^5^ injection of cells (n = 6–8/group) on day 0 to assess **(D)** survival (Log-Rank test, **p ≤ 0.01), or **(E)** tumour progression, and **(F)** tumour burden on day 14**.** Tumours measured approximately every 2 days using calipers to determine tumour volume ((W × W × L)/2). Significance determined by Student’s t test, **p ≤ 0.01. Data are representative of 2–3 independent experiments.

We first sought to determine whether the absence of FGL2 (*Fgl2*^*−/−*^ mice) relieved immunosuppression and prolonged survival in melanoma and ovarian cancer models. To specify the role of immune/stromal cell-produced FGL2, we took advantage of *Fgl2*-deficient mice and injected them with either ovarian cancer (ID8-*p53*^*−/−*^*Brca2*^*−/−*^) or melanoma (B16F10) cells. These mouse cancer cell lines do not express FGL2 in vitro (undetected by qPCR and ELISA) nor in vivo (Fig. S4A), in agreement with human ovarian cancer and melanoma (Fig. 1). The absence of FGL2 did not prolong survival in i.p. ID8*-p53*^*−/−*^*Brca2*^*−/−*^ tumour-bearing mice (Fig. 2A). However, when we assessed tumour burden at a late timepoint (day 46, immediately prior to the day mice began reaching endpoint) in the orthotopic (i.b.) ID8*-p53*^*−/−*^*Brca2*^*−/−*^ model, primary tumour burden was significantly lower in the absence of FGL2 (Fig. 2B). Additionally, we collected all peritoneal metastatic tumour nodules and found a lower total mass of metastases (Fig. 2C), with 4/5 *Fgl2*^*WT*^ mice developing metastases, but only 1/6 *Fgl2*^*−/−*^ mice had metastases. To examine the possibility that genetic modification of the ID8 cell line (deleting *Trp53*, *Brca2*) might affect tumour progression, we similarly assessed survival of an i.p. model of ID8 ovarian cancer; however the absence of FGL2 did not prolong survival in this model either (Fig. S4B).

In parallel, we assessed how FGL2 may influence tumour development of a more immunogenic tumour model, B16F10. The absence of FGL2 significantly prolonged survival in mice injected with B16F10 s.c. (Fig. 2D) as well as significantly delayed tumour progression (Fig. 2E). Primary tumour burden in these mice at a single timepoint, again immediately prior to endpoint (day 14) was significantly lower in the absence of FGL2 (Fig. 2F). To account for potential differences due to location of tumour inoculation, we injected the B16F10 cells i.p. and assessed survival. Similar to the s.c. model, the absence of FGL2 significantly prolonged survival (Fig. S4C). As absence of FGL2 reduced tumour burden in both models, we next sought to determine whether FGL2 normally enhances tumour progression by inducing immune cell changes in the TME.

### FGL2 is associated with lower expression of NK activation markers and fewer tumour infiltrating NK cells

**Figure 3:**
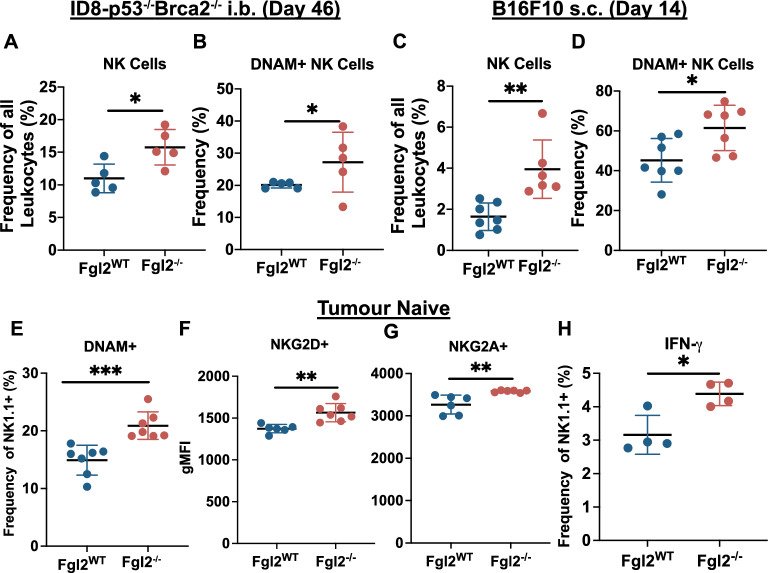
The absence of FGL2 in the tumour microenvironment increases infiltration of activated NK cells.* Fgl2*^*WT*^ and *Fgl2*^*−/−*^ mice received bilateral i.b. injections of 0.15 × 10^6^ ID8-*p53*^*−/−*^*Brca2*^*−/−*^ cells (n = 5/group) or s.c. injections of 2 × 10^5^ B16F10 cells (n = 7/group). Frequency of NK cells (**A,C**) and DNAM-1 + NK cells (**B,D**) in ID8-*p53*^*−/−*^*Brca2*^*−/−*^ tumours collected on day 46 and B16F10 tumours (day 14) were assessed by flow cytometry. Spleens of tumour-naïve *Fgl2*^*WT*^ and *Fgl2*^*−/−*^ mice (n = 7/group) were assessed by flow cytometry to assess frequency of **(E)** DNAM-1+ NK cells, geometric mean florescence intensities (gMFI) of **(F)** NKG2D+ and (**G)** NKG2A+ in NK cells and frequency of **(H)** IFN-γ+ NK cells. Gating strategy: minus debris/singlets/live cells/CD45+, NK Cells (NK1.1+ CD3−) and DNAM-1+, IFN-y+, NKG2D+, NKG2A+, NK cells. Significance determined by Student’s t test. *p ≤ 0.05, **p ≤ 0.01, ***p ≤ 0.001.

FGL2 has been associated with immunosuppressive immune cell populations and with diminished activation/maturation of T cells and DCs^12,25,27,28,31^. To determine if FGL2 favoured tumour progression by inhibiting anti-cancer immunity, we characterized immune cell infiltration in B16F10 or ID8-*p53*^*−/−*^*Brca2*^*−/−*^ tumours implanted into *Fgl2*^*−/−*^ mice. Flow-cytometric analysis (Fig. S5) was performed on tumours and spleens collected at day 46 for i.b. ID8-*p53*^*−/−*^*Brca2*^*−/−*^ tumour-bearing mice and day 14 for s.c. B16F10 tumour-bearing mice. In *Fgl2*^*−/−*^ mice we observed a higher infiltration of NK cells in both ID8-*p53*^*−/−*^*Brca2*^*−/−*^ (Fig. 3A) and B16F10 primary tumours (Fig. 3C). Furthermore, in the absence of FGL2 the frequency of NK cells expressing DNAM-1+ (DNAX accessory molecule, CD226), considered to be more activated and cytotoxic^44^, was substantially higher (Fig. 3B,D). While both tumour-bearing and tumour-naïve mice had a lower frequency of NK cells in the spleen (Fig. S6A–C), a higher frequency of DNAM-1+ NK cells in *Fgl2*^*−/−*^ mice was observed not only in the tumours, but also in the spleen of tumour-naïve animals (Fig. 3E). Higher expression of NKG2D and NKG2A (Fig. 3F, G) suggested that, in the absence of FGL2, NK cells are more activated even in the absence of a pathological challenge. Interestingly, when we probed the basal expression of IFN-γ in resting NK cells, lack of FGL2 resulted in a small but consistent increase in cytokine accumulation (Fig. 3H), supporting the idea that FGL2 suppresses NK cell activation^44–47^. However, higher activation in the absence of FGL2 did not translate to an advantageous response to re-stimulation, as we assessed by stimulating *Fgl2*^*WT*^ and *Fgl2*^*−/−*^ splenic NK cells with plate-bound antibodies and chemical activators in CD107 (LAMP1) degranulation, or target cells (YAC1) (Fig. S6D, E). In all instances, the cytotoxic response of NK cells was similar when stimulating *Fgl2*^*WT*^ and *Fgl2*^*−/−*^ NK cells, which indicates that FGL2 inhibition of NK cell activation can be overcome by acute stimulation, and further suggesting that FGL2 acts as a physiological checkpoint to NK cell activity. Given the higher activation of NK cells in *Fgl2*^*−/−*^ mice, we reasoned that the survival advantage in *Fgl2*^*−/−*^ mice could be due to improved NK cell anti-cancer activity. However, depleting NK cells in vivo in the s.c. B16F10 model did not significantly hamper the effect of the absence FGL2 in prolonging survival (Fig. S6F). Therefore, while NK cells in the absence of FGL2 have significantly higher markers of NK activation by flow cytometry, they do not significantly contribute to prolonged survival or reduced tumour burden in *Fgl2*^*−/−*^ mice, suggesting that FGL2 had some NK cell-independent effects.

### Absence of FGL2 is associated with more activated dendritic and T cells in the TME

**Figure 4:**
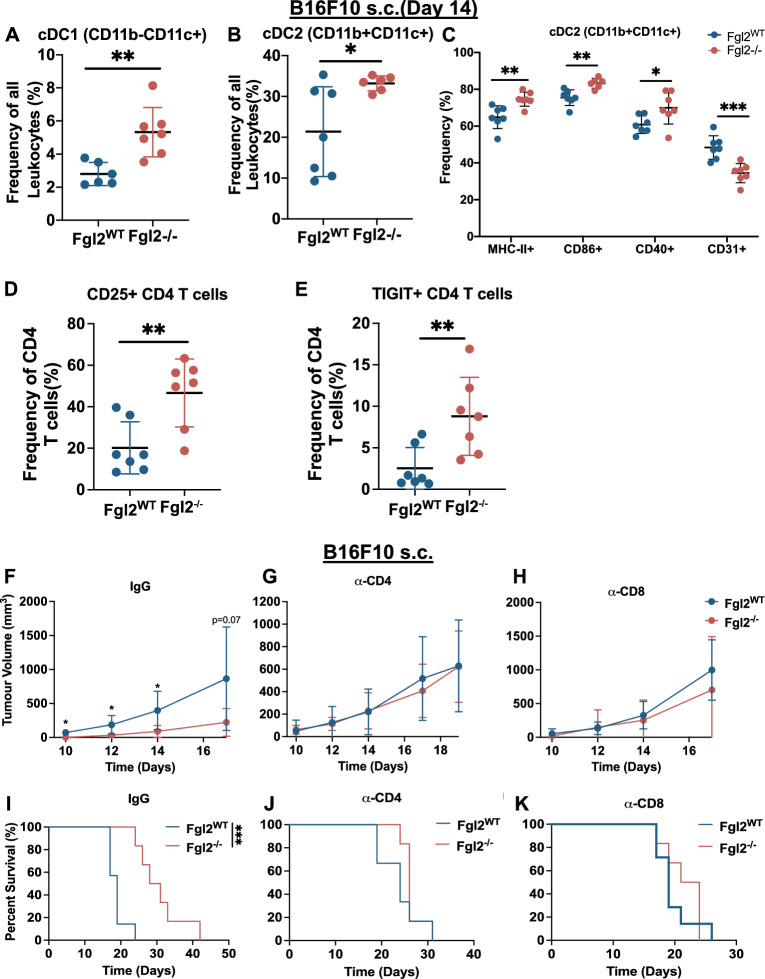
The absence of FGL2 in the tumour microenvironment increases activated DCs and T cells in B16F10 tumours. *Fgl2*^*WT*^ and *Fgl2*^*−/−*^ mice received a s.c. injection of 2 × 10^5^ B16F10 cells (n = 7/group) on day 0. Tumours were collected on day 14 and total frequency of cDC1s **(A)** and cDC2s **(B)** with the frequency of MHC-II+, CD86+, CD31+, and CD40+ cDC2s **(C)** were assessed by flow cytometry. Gating strategy: minus debris/singlets/live cells/CD45+, followed by: cDC1s (CD3− CD11b− CD11c+), cDC2s (CD3− CD11b+ CD11c +), MHC-II, CD86, CD40, and CD31 frequency. Frequency of **(D)** CD25+ and **(E)** TIGIT+ CD4+ T cells were assessed in B16F10 tumours. Gating strategy: minus debris/singlets/live cells/CD45+, T cells (NK1.1− CD3+) and CD4+ CD8−, and CD25+, TIGIT+ frequency. Significance determined by Student’s t test. *p ≤ 0.05, **p ≤ 0.01. **(F–K)** *Fgl2*^*WT*^ and *Fgl2*^*−/−*^ mice** (**n = 6mice/group) were treated 500 μg/mouse IgG control antibody **(F,I)** or anti-CD4 **(G,J)** or 250 μg/mouse anti-CD8 **(H,K)** to deplete T cells one and two days before tumour injections (B16F10 2 × 10^5^ cells s.c.) followed by twice weekly injections of 200 μg/mouse of depletion antibodies. **(F–H)** Tumour progression was measured approximately every 2 days using calipers to determine tumour volume ((W × W × L)/2). Significance determined by Student’s t test, *p ≤ 0.05. **(I–K)** Kaplan–Meier plots showing survival. Significance determined by Log-Rank test, ***p ≤ 0.001.

DCs play a key role in the generation of anti-tumoural responses and are key targets of FGL2-mediated immunosuppression^3,25,28^. We therefore sought to profile the effect of FGL2 absence on tumour-infiltrating and splenic DCs in B16F10 and ID8-*p53*^*−/−*^*Brca2*^*−/−*^ tumours. In the B16F10 model, there was a greater frequency of tumour-infiltrating DCs in *Fgl2*^*−/−*^ mice, both cDC1s (Fig. 4A) and cDC2s (Fig. 4B). cDC2s expressed more co-stimulatory markers (MHC-II+, CD86+, CD40+) and had a decreased frequency of the co-inhibitory marker CD31 (Fig. 4C), suggesting greater antigen presentation capabilities and priming of CD4+ T cells, responses not seen in cDC1s (Fig. S7A). Conversely, in ID8-*p53*^*−/−*^*Brca2*^*−/−*^ tumours, we found increased cDC1s (Fig. S7B) and fewer cDC2s (Fig. S7C) in *Fgl2*^*−/−*^ mice, with no significant differences in co-stimulatory marker expression (Fig. S7D, E). No difference was observed in total splenic DC frequencies (Fig. S7F–H) in tumour-bearing or tumour-naïve mice, but we observed increased expression of co-stimulatory markers (MHC-II+, CD86+, CD40+) (Fig. S7I) in *Fgl2*^*−/−*^ spleens of ID8-*p53*^*−/−*^*Brca2*^*−/−*^ tumour-bearing mice, but not B16F10 (Fig. S7J). This is similar to what is observed in tumour-naïve *Fgl2*^*−/−*^ mice with greater expression of co-stimulatory marker (CD86+) (Fig. S7K), as previously reported^5^, suggesting more antigen presentation in the periphery. Overall, we show that B16F10 tumours were infiltrated by more DCs and more activated DCs, a characteristic of *Fgl2*^*−/−*^ mice^3^, which may contribute to tumour elimination and prolonged survival in this model. This was not the case in the ID8*-p53*^*−/−*^*Brca2*^*−/−*^ model, as DCs of *Fgl2*^*−/−*^ mice with higher expression of co-stimulatory molecules were found only in the spleen.

We further performed some broad characterization of the myeloid compartment where CD11b+ cells were decreased in ID8-*p53*^*−/−*^*Brca2*^*−/−*^ tumours (Fig. S8A), but increased in B16F10 tumours in *Fgl2*^*−/−*^ mice (Fig. S8B). No difference was detected in CD11b+ Gr1+ cells in either the ID8-*p53*^*−/−*^*Brca2*^*−/−*^ or B16F10 tumours (Fig. S8C, D). Due to limited tumour tissue in the ID8-*p53*^*−/−*^*Brca2*^*−/−*^ model, we did not perform further characterization of the myeloid compartment, but interestingly there were increased Ly6C+ cells (Fig. S8E), including inflammatory monocytes (F480+ MHC-II−) (Fig. S8F) and macrophages (F480+ MHC-II+) (Fig. S8G) in B16F10 tumours in *Fgl2*^*−/−*^ mice, again suggesting that the absence of FGL2 promotes a TME primed for pro-inflammatory tumour targeting and elimination.

T cells, particularly CD4+ T cells, have been shown to be a downstream target of FGL2-mediated immunosuppression of DCs^5,17,18,20,23^. Given that *Fgl2*^*−/−*^ DCs have more co-stimulatory molecules, we expected that the absence of FGL2 would correspond to more T cell activation and therefore we characterized tumour-infiltrating T cells and splenic T cells in B16F10 or ID8-*p53*^*−/−*^*Brca2*^*−/−*^ tumours implanted into *Fgl2*^*WT*^ and *Fgl2*^*−/−*^ mice. In the absence of FGL2 we observed increased T cells (CD3+) overall in ID8*-p53*^*−/−*^*Brca2*^*−/−*^ tumours (Fig. S9A) and the CD4+ T cells showed a trend (p = 0.06) for higher expression of TIGIT (Fig. S9B). Although we observed no significant difference in T cells in B16F10 tumours in *Fgl2*^*−/−*^ mice, there was a trend towards decreased T cells (p = 0.0534) (Fig. S9C), but the response appears bimodal. Interestingly, CD4 T cells in B16F10 tumours in *Fgl2*^*−/−*^ mice were more CD25+ (Fig. 4D) and TIGIT+ (Fig. 4E). Similarly, we observed a trend (p = 0.0504) for more CD25+ CD8+ T cells (Fig. S9D). While CD25 and TIGIT indicates activation of T cells has taken place through TCR engagement, often these are markers of exhausted T cells^48–50^. Overall, the absence of FGL2 leads to increased frequency of T cells in ID8-*p53*^*−/−*^*Brca2*^*−/−*^ tumours and increased CD25+ and TIGIT+ CD4 T cells in B16F10 tumours.

Given the increase in B16F10 tumour-infiltrating DCs, co-stimulatory molecules on DCs, and activated (CD25+, TIGIT+) CD4 T cells, we predicted DC and T cell interactions might play a role in prolonging survival in *Fgl2*^*−/−*^ mice. Therefore, we depleted either CD4 and CD8 T cells in vivo in the s.c. B16F10 model and assessed tumour progression and survival. In mice treated with an IgG control antibody, as expected, tumour progression in *Fgl2*^*−/−*^ mice was slower compared to *Fgl2*^*WT*^ mice (Fig. 4F; Fig. S9E). In contrast, when either CD4 or CD8 T cells were depleted, tumour progression occurred similarly in both *Fgl2*^*WT*^ and *Fgl2*^*−/−*^ mice (Fig. 4G, H; Fig. S9F–H). Additionally, when treated with an IgG control antibody, *Fgl2*^*−/−*^ mice survived longer than *Fgl2*^*WT*^ mice, as expected (Fig. 4I), whereas survival was similar when either CD4 or CD8 T cells were depleted (Fig. 4J, K). Therefore, CD4 and CD8 T cells were essential to the reduced tumour burden, slower tumour progression, and prolonged survival observed in *Fgl2*^*−/−*^ mice.

### Fgl2 absence and oncolytic virotherapy synergize to prolong survival of ovarian tumour-bearing mice

**Figure 5:**
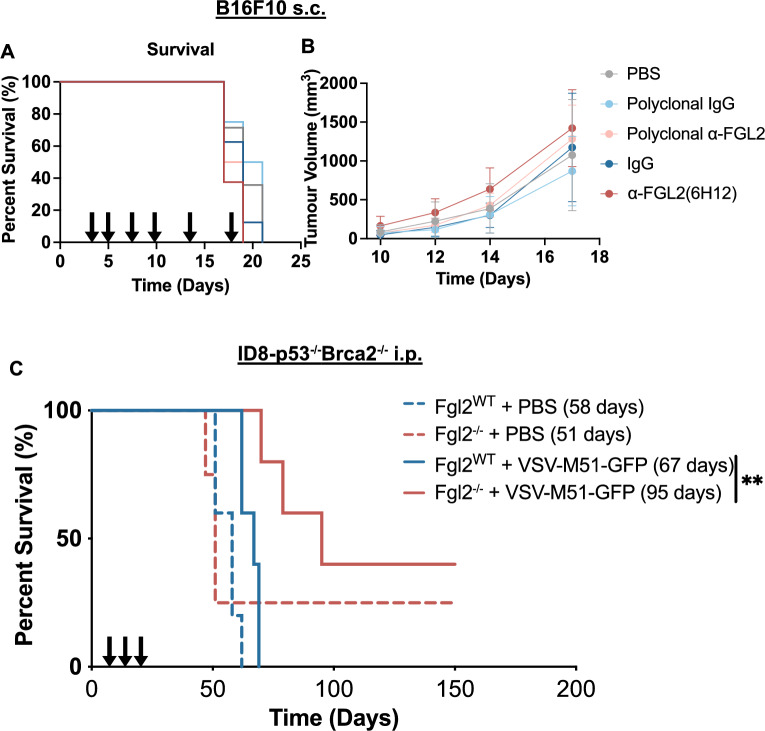
The absence of FGL2 combined with oncolytic virus treatment synergizes to prolong survival of ID8*-p53*^*−/−*^*Brca2*^*−/−*^ tumour-bearing mice. Mice were injected s.c. with 2 × 10^5^ B16F10 cells (8 mice/group) on day 0 and were treated with α-polyclonal FGL2 (100 μg/mouse) or α-monoclonal FGL2 (6H12) (150 μg/mouse) antibodies starting 3 days after tumour cell injections, followed by injections on day 5 and 7, and continued 2×/week (arrows). Mice assessed for **(A)** survival and **(B)** tumours were measured every 2–3 days. **(C)** *Fgl2*^*WT*^ and *Fgl2*^*−/−*^ mice received an i.p. injection of 5 × 10^6^ ID8*-p53*^*−/−*^*Brca2*^*−/−*^ cells on day 0 followed by VSV-M51-GFP treatment injected i.p. on day 7, 10, and 13 (arrows). (n = 5/group). Mice were assessed for survival. Significance determined by Log-Rank test, **p ≤ 0.01. Median survival is indicated in parentheses.

Given that the absence of FGL2 prolonged survival in the B16F10 model, we next sought to therapeutically target FGL2 with a blocking antibody. Based on our observations of increased infiltration of activated NK cells, T cells and DCs into the tumour, we focused on assessing the effects of FGL2 deficiency in the context of sFGL2 action on tumour infiltrating lymphocytes. However, it should be noted that we did observe decreased CD31 + (endothelial) cells in the B16F10 tumours implanted in *Fgl2*^*−/−*^ mice (Fig. S10A), suggesting the absence of FGL2 also decreased vascularization of the tumour. Decreased vascularization in the TME has previously been attributed to the prothrombinase function of mFGL2^34,36^. Therefore, we sought to distinguish the consequences of targeting both forms of FGL2 using a polyclonal α-FGL2 antibody^2^ from the effects of a sFGL2-blocking monoclonal antibody (clone 6H12)^51^ in the B16F10 model. Surprisingly, neither the monoclonal nor the polyclonal antibody prolonged survival compared to their appropriate IgG controls (Fig. 5A), nor did they slow the rate of tumour progression (Fig. 5B).

We next used the STOSE ovarian cancer model, as a more immunogenic model (expresses MHC-I in vivo) with a greater chance of responding to immunotherapy^52^, and targeted sFGL2 with the monoclonal α-FGL2 (clone 6H12). Blocking FGL2 did not prolong survival when compared to the appropriate IgG control or PBS control (Fig. S10B). In this model, we assessed FGL2 levels in the plasma following 7 days of treatment (Fig. S10C) and in the ascites at endpoint (Fig. S10D), demonstrating that the antibody successfully targeted sFGL2. However, the splenic immune cell populations after 7 days of treatment showed increased activation (CD25+, TIGIT+) of CD4+ T cells (Fig. S10E) when FGL2 activity was blocked, similar to what was observed in *Fgl2*^*−/−*^ mice bearing B16F10 and ID8-*p53*^*−/−*^*Brca2*^*−/−*^ tumours.

“Cold” tumours present a challenge for immunotherapy success, including low MHC-I expression, lack of tumour-associated antigens, and/or poor immune cell infiltration into the tumour^53^. Considering that in the absence of FGL2, DCs in the spleens of ID8-*p53*^*−/−*^*Brca2*^*−/−*^ tumour-bearing mice were more activated, but were excluded from the tumour, we therefore combined immunotherapeutic strategies in order to convert the “cold” tumour (MHC-I low^54^, excluded activated DC) into a “hot” tumour^55,56^. Oncolytic virotherapy is an immunotherapeutic strategy being tested in several clinical trials, and has been shown to turn a “cold” tumour into a “hot” tumour^53,57,58^. Oncolytic viruses, such as recombinant vesicular stomatitis virus (VSV), selectively infect and kill cancer cells as well as induce immunogenic cell death, stimulating immune cell infiltration and activation of antigen-presenting cells (DCs and macrophages)^59,60^. ID8-*p53*^*−/−*^*Brca2*^*−/−*^ cancer cells were injected i.p., followed by treatment with 1 × 10^8^ pfu of VSV-M51-GFP^59^ oncolytic virus on days 7, 10, and 13. While neither the absence of FGL2 alone (median survival 51 days) nor the oncolytic virus alone (median survival 67 days) were able to prolong survival compared to untreated *Fgl2*^*WT*^, the combination treatment synergized to prolong survival (median survival 95 days, Log-Rank test, **p ≤ 0.01) (Fig. 5C), with two mice surviving past 150 days, as well as one mouse in the *Fgl2*^*−/−*^ group which received PBS. Surviving mice were re-challenged with 3 × 10^6^ ID8*-p53*^*−/−*^*Brca2*^*−/−*^ cells on day 150, but all reached endpoint as expected (around 55 days post-rechallenge), suggesting no memory response resulted from the combination of *Fgl2*^*−/−*^ and oncolytic virus (VSV-M51-GFP) (Fig. S10G). Overall, we found that blocking FGL2 alone was insufficient to prolong survival as a novel immunotherapy for ovarian cancer. However, when treating a model that was previously unresponsive to the absence of FGL2 with an oncolytic virus, the combination significantly prolonged survival. This suggests that loss or blockage of FGL2 combined with other immunotherapies may set the stage to enhance response to certain immunotherapies.

## Discussion

FGL2 expression has been associated with poor survival in a number of different cancers such as glioblastoma multiforme^28,30,31^, colorectal^32^, renal^33^, liver^25,34,35^, and prostate cancer^36^ primarily through its prothrombinase function, as well as a mediator of immunosuppression. By querying scRNA-seq data of many human cancer types, including ovarian, melanoma, lung, breast and colorectal, we have shown that FGL2 is not expressed in cancer cells, but rather multiple immune and stromal cell types in the TME, with predominant expression in monocytes and macrophages. sFGL2 levels are higher in human ovarian cancer ascites than non-cancer ascites, and have low levels of proinflammatory cytokines, suggesting sFGL2 contributes to the immunosuppressive TME in ovarian cancer. Further, we and others^25^ showed that FGL2 expression in non-cancer cells in the TME is associated with poor survival, with larger tumour burden and faster tumour progression, as well as suppression of activation markers on T cells, DCs, and NK cells.

**Figure 6:**
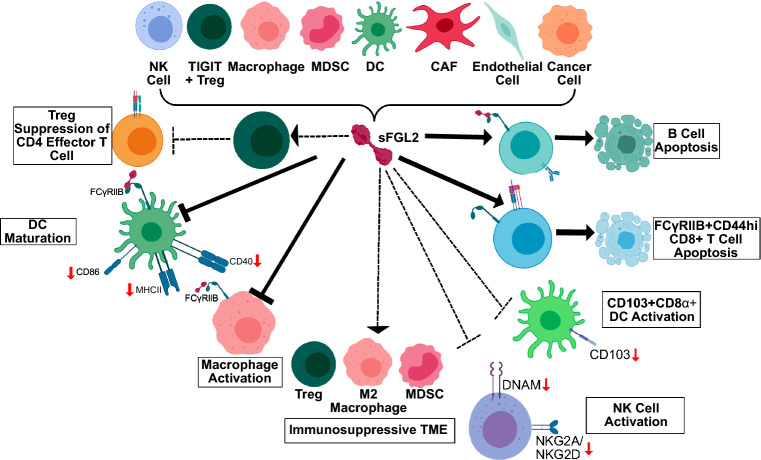
FGL2 is expressed in macrophages, T cells (notably Tregs/TIGIT+ Tregs), endothelial cells, cancer associated fibroblasts (CAFs), natural killer (NK) cells, dendritic cells (DCs), and myeloid-derived suppressor cells (MDSCs). sFGL2 acts directly (solid line) to suppress DC maturation (CD80, CD86, MHC-II downregulation) and macrophage activation. Further contributing to immunosuppression, sFGL2 can directly induce B cell and FCγRllB+ CD44hi CD8+ T cell apoptosis. sFGL2 is associated (dotted line) with enhanced Treg suppression of effector T cells, suppression of CD103+ CD8a+ DC activation, and with populations of immunosuppressive immune cell populations (Tregs, M2 macrophages, and MDSCs). We show for the first time the association between sFGL2 and suppression of NK cell activation markers.

We demonstrated for the first time that *Fgl2*^*−/−*^ mice have more DNAM-1+ NK cells and higher expression of NKG2D+ and NKG2A+ markers (Fig. 6) and that these DNAM-1+ NK cells are found at higher frequencies in both ovarian (ID8*-p53*^*−/−*^*Brca2*^*−/−*^) and melanoma (B16F10) tumours. Ostapchuk et al.^61^ proposed that FGL2 directly contributes to suppression of NK cell cytotoxicity while Yu et al.^7^ suggested *Fgl2* expression in NK cells is associated with a subtype of NK cell with impaired cytotoxicity. In our study, no functional differences were observed between tumour-naïve NK cells isolated from *Fgl2*^*WT*^ and *Fgl2*^*−/−*^ mice either by using a CD107a+ degranulation assay as a readout of NK cell activation and cytotoxicity^62^, or by direct NK-mediated target cell lysis. NK cells may be functionally different via other pathways such as granzyme production, proliferation, or ADCC^63^ and may be investigated in future studies. However, depleting NK cells in vivo did not suggest NK cells were a driving factor to prolong survival in *Fgl2*^*−/−*^ mice. In the initial characterization of *Fgl2*^*−/−*^ mice^5^, differences in NK cells were not investigated, but *Fgl2*^*−/−*^ mice do develop autoimmune glomerulonephritis which is attributed to impaired Treg activity. Therefore, NK cell marker expression may be a downstream consequence of more activated DCs and CD4+ T cells in *Fgl2*^*−/−*^ mice, given activated DCs and T cells produce more IL-15 and IL-2, inducing NK cell marker expression^64^. In one study, FGL2 suppressed MCP-1 secretion from macrophages which in turn lessened NK cell recruitment and IFN-γ production^16^, suggesting other possible downstream mechanisms of FGL2 targeting NK cells and their decreased activation.

Given FGL2’s association with several immunosuppressive cell populations (M2 macrophages, Tregs, MDSCs)^12,19,27,31^ and its role in suppression of DC and T cell activation^3,5,6,25^, we performed a broad characterization of these immune cell populations in order to narrow down the role of FGL2 in our two model systems. There were more T cells in the tumours of ID8*-p53*^*−/−*^*Brca2*^*−/−*^ mice and more activated T cells and cDC2s in the B16F10 tumours, similar to findings by Yang et al.^25^ who investigated the effect of FGL2 produced by cells in the TME of hepatocellular carcinoma models. Those authors demonstrated that sFGL2 acts directly on DCs (CD11c+) to suppress activation (CD40, CD86, and MHCII), subsequently suppressing T cell activation, by reduced phosphorylation of Akt, NF-κB, CREB, and p38 in DCs^25^. They also observed a downregulation of CD31 in DCs in the absence of FGL2, similar to the decrease in CD31 we observed in DCs of the B16F10 tumours. CD31 acts as a co-inhibitory receptor, where CD31+ DCs are more tolerogenic, suggesting in the absence of FGL2 cDC2s in B16F10 tumours are more activated (MHCII+, CD86+, CD40+) and less immunosuppressive (CD31+). However, we also observed increased expression of this immune checkpoint in spleens of *Fgl2*^*−/−*^ tumour-naïve and ID8*-p53*^*−/−*^*Brca2*^*−/−*^ tumour-bearing mice, suggesting a negative feedback mechanism to keep the more activated pro-inflammatory DCs in check in the absence of FGL2^65^. In one study, FcγR engagement led to the downregulation of CD31, which may prompt further studies as to whether sFGL2 is directly acting on DCs through the Fc receptors to downregulate CD31^66^.

Knocking out FGL2 or blocking the function of sFGL2 via monoclonal antibody treatment led to increased expression of CD25 and TIGIT on CD4+ T cells, but did not prolong survival. Although the concentration of antibody used has previously been shown to be effective in vivo ^2,51,67^, and we show significant targeting of FGL2 in the plasma following treatment and in the ascites, we did not conduct dose titration studies, so it is possible that the inability of α-FGL2 to enhance survival or reduce tumour burden might be due to low intra-tumoural concentration of the antibody. However, in mouse models of glioblastoma^28,30,31^ and hepatocellular carcinoma^25^ 100 μg/mouse was used to prolong survival, which is less than our dose of 150 μg/mouse. This further suggests the model specificity of the role of FGL2 and that other factors may play a dominant role. Furthermore, although expression of CD25 and TIGIT on CD4 T cells suggests that the T cell population has been activated through TCR engagement^48,49^, CD25+ FOXP3+ or TIGIT+ T cells typically represent a more immunosuppressive or regulatory T cell population^27,68^. Unlike other models which appear to have decreased frequencies of Tregs in the absence of FGL2^25,30,31^, we observed greater frequencies of CD25+, TIGIT+ CD4+ T cells, suggesting an increased abundance of Tregs. In the B16F10 and STOSE models, where blocking FGL2 did not prolong survival, increased presence of TIGIT+ Tregs, which do express high amounts of FGL2^27^, may have impeded tumour elimination. Therefore, TIGIT checkpoint blockade in combination with FGL2 blocking antibody therapy may be more successful in prolonging survival^69^.

While FGL2 appears to be involved in numerous common immune pathways in the TME, the response to the absence of FGL2 is clearly model-specific. In this study, only B16F10 tumour-bearing mice had prolonged survival in the absence of FGL2. Whereas smaller tumour burden was observed at a single timepoint for ID8*-p53*^*−/−*^*Brca2*^*−/−*^ tumour-bearing mice, this did not translate to prolonged survival, which may be due to the development of ascites which happens quickly at later stages of tumour progression and drives loss of wellness at endpoint. Both B16F10 and ID8*-p53*^*−/−*^*Brca2*^*−/−*^ cell lines express no/low MHC-I and are poorly immunogenic^54,70^. However, B16F10 cells do express immunogenic antigens such as GP100 or TRP-2^71,72^; in contrast, it is known that the parental ID8 cell line does not express any immunogenic antigens^73^. Immunogenic antigens present in B16F10 cells may explain why the DCs and T cells in B16F10 tumours are more activated. Depleting CD4+ and CD8+ T cells in *FGL2*^WT^ versus *Fgl2*^*−/−*^ B16F10 tumour-bearing mice showed the dependence on both CD4 and CD8 T cells for prolonged survival in *Fgl2*^*−/−*^ mice, which further supports the involvement of the adaptive immune system and importance of a strong antigenic response in the role of FGL2-mediated tumour progression. ID8*-p53*^*−/−*^*Brca2*^*−/−*^ tumours may have differences in the extracellular matrix, vascularization, or in cytokine/chemokine expression, thereby limiting immune cell infiltration into the tumour^74^ since more activated DCs and T cells were observed systemically, i.e. in the spleen of tumour-bearing mice. Further studies to investigate the relationship between the presence of strong antigens and the role of FGL2 may help us to understand the biological role of FGL2 and why there are such different model-dependent results. Determining the role of strong antigens in FGL2 immunomodulation will further clarify the clinical feasibility of targeting FGL2. Therefore, it is remarkable that a poorly immunogenic cancer model which did not respond to a complete knockout in FGL2 (*Fgl2*^*−/−*^ mice) was able to survive significantly longer with the addition of an oncolytic virus to an FGL2-deficient background. This may suggest that the oncolytic virus stimulated immune cell trafficking and immunogenic cell death in the primary tumours^60^, allowing the more activated DCs found in the *Fgl2*^*−/−*^ spleens to traffic to the tumour, thereby prolonging survival. Future studies are required to determine whether blocking FGL2 using monoclonal antibody therapy can also synergize with oncolytic virus therapy.

While knocking out/blocking FGL2 has subtle effects on altering immune cell phenotypes in the TME, resulting in prolonged survival in only some models of cancer, the results of this study show that it can potently synergize with oncolytic virus immunotherapy to successfully prolong survival in a model that is primed by the absence of FGL2. In clinical trials to treat ovarian cancer, immune checkpoint inhibitors have had limited success with response rates of 5–20%^75^, highlighting the need to develop novel immunotherapeutic strategies. While immune checkpoint blockade in melanoma has had marked success, resistance is still a challenge in the treatment of melanoma^76^. We propose that targeting FGL2 in the TME has the potential to be another useful tool to incorporate into combination immunotherapies.

## Materials and methods

### Primary cells and cell lines

ID8 cells were generated by Roby et al.^77^ and ID8-*p53*^*−/−*^-*Brca2*^*−/−*^ cells were provided by Dr. Ian McNeish ^78^. Cells were maintained in DMEM + 4% fetal bovine serum (FBS, Hyclone) and 0.01 mg/mL insulin–transferrin–sodium–selenite solution (ITSS; Roche). STOSE cells were generated by our lab^79^ and maintained in α-MEM + 4% FBS with ITSS and 2 μg/ml epithelial growth factor (EGF, R&D systems). B16F10^80^ cells were provided by Dr. Rebecca Auer (Ottawa Hospital Research Institute, OHRI) and were maintained in DMEM+ 10% FBS. Splenic NK cells and YAC-1 cells^81^ (provided by Dr. Michele Ardolino, OHRI) were maintained in complete NK media (RPMI+ 10% FBS, 1% penicillin/streptomycin, 10 mM HEPES, 55 μM 2-mercaptoethanol). Mycoplasma testing was performed prior to animal experiments. Cells were incubated at 37 °C with 5% carbon dioxide.

### Mice and in vivo mouse models of cancer

Animal experiments were carried out using protocols approved by the Animal Care Committee at the University of Ottawa and conforming to the standards defined by the Canadian Council on Animal Care. This study was conducted in compliance with the Animal Research: Reporting of In Vivo Experiments (ARRIVE) guidelines. *Fgl2*^*WT*^ (C57BL/6) mice were bred from mice purchased from The Jackson Laboratory. *Fgl2* knockout (*Fgl2*^*−/−*^) mice, harbouring a complete knockout of *Fgl2* and therefore lacking both mFGL2 and sFGL2, were generated by Dr. Gary Levy^1^. For all experiments, mice were used at 8–10 weeks of age, age-matched between *Fgl2*^*WT*^ and *Fgl2*^*−/−*^. For intraperitoneal (i.p.) injections mice received either 2.5 × 10^4^ of B16F10 melanoma cells or 5 × 10^6^ ovarian cancer cells (ID8 or ID8-*p53*^*−/−*^*Brca2*^*−/−*^) in 100 μl PBS. In a bilateral orthotopic intrabursal (i.b) model of ovarian cancer 1.5 × 10^5^ ID8-*p53*^*−/−*^*Brca2*^*−/−*^ cells were injected under each ovarian bursa in 2 μl PBS, as previously described^79^. For subcutaneous (s.c.) injection of B16F10 melanoma cells, 2 × 10^5^ cells were injected into the right flank of the mouse in a volume of 100 μl PBS. Subcutaneous tumours were measured using calipers approximately every two days and tumour volume was calculated using the formula (width × width × length)/2, where width (W) is the shortest diameter and length (L) is the longer. FVB/N mice were purchased from Charles River and received an i.p. injection of 5 × 10^6^ STOSE ovarian cancer cells in 100 μl PBS. In all cases mice were assessed for survival until humane endpoint, except where tumours (primary and metastases) and spleens were collected for flow cytometry analysis at the indicated timepoints.

### T and NK cell depletion

To deplete NK cells 200 μg α-NKR-P1C (clone PK136, Leinco Technologies) or IgG control antibody (Leinco Technologies) was injected i.p. on days –1 and –2 prior to tumour inoculation followed by once per week depletions. To deplete T cells 500 μg α-CD4 (clone GK1.5, Leinco Technologies) or 250 μg α-CD8 (Clone Ly2.2) or IgG control antibody (Leinco Technologies) was injected i.p. on days –1 and –2 prior to tumour inoculation followed by 200 μg twice per week depletions. T and NK depletion was verified by flow cytometry of spleens at endpoint.

### FGL2 antibody treatment

To block FGL2, the monoclonal α-FGL2 (6H12) and the polyclonal α-FGL2 were generously provided by Dr. Gary Levy^2,51,67^. The mouse IgG_1_ isotype control (clone HKSP) for monoclonal α-FGL2 (6H12) was purchased from Leinco Technologies. A polyclonal IgG control was purified from rabbit serum using Protein A Columns (Thermo Scientific™). For all experiments daily injections of 150 μg/mouse of the monoclonal α-FGL2 or 100 μg/mouse of the polyclonal α-FGL2 or their respective IgG control was injected i.p. in 100 μL PBS. For B16F10 s.c. tumour-bearing mice, treatment began on day 3 after tumour inoculation followed by treatment on day 5 and 7 and then twice per week (day 10, 14, 17). For STOSE i.p. tumour-bearing mice, treatment began 31 days post tumour cell injection, and mice received one daily injection for 7 days.

### Oncolytic virus treatment

VSV-M51-GFP is a recombinant virus with a deletion of methionine 51 in the M protein and encoding GFP^59^. The virus was kindly provided by Drs. John Bell and David Stojdl (OHRI)^59^. The virus was propagated in Vero cells, purified by ultra-centrifugation, and quantified by standard plaque assay, as previously described^82^. Mice received 1 × 10^8^ pfu VSV-M51- GFP in 100μL PBS by i.p. injection on days 7, 10, and 13 post tumour cell injection.

### ELISA and LEGENDplex™ assays

Plasma derived from blood collected by cardiac puncture into heparin coated capillary tubes (Microvette CB 300 Lh, Sarstedt) and ascites fluid were acquired from mice bearing STOSE tumours, centrifuged at 2000×*g* for 15 min, and supernatant was frozen at −80 °C until assay. Human ascites supernatants (Supplemental Table 1) were acquired from the Ottawa Ovarian Cancer Tissue Bank (Ottawa, Canada) with Research Ethics Board approval (Protocol 1999540-01H) and informed consent and were frozen at −80 °C until assay. For mouse and human FGL2 ELISA (BioLegend), ascites samples were diluted 1:10 and assayed according to manufacturer’s instructions. Notably, the BioLegend kits use the FGL2 antibodies generated by Dr. Levy.

For LEGENDplex assays, human ascites samples were diluted 1:2 in Assay Buffer and assayed according to manufacturer’s protocol for serum/plasma samples with 3 multiplex bead-based assays: LEGENDplex™ Human Cytokine Panel 2 (13-plex), Human Proinflammatory Chemokine Panel (13-plex), and Human Inflammation Panel 1 (13-plex) (BioLegend). Samples were acquired in duplicate the same day of staining, on a BD LSR FortessaTM flow cytometer and analyzed using LEGENDplex Quognit software (BioLegend).

### Flow cytometry

Samples were prepared for flow cytometry as described previously^54^. Briefly, single cell suspensions were generated from tumours using the mouse Tumour Dissociation Kit (Miltenyi) and splenocytes by mechanical dissociation. Cells were stained for viability (FVS510, BD Biosciences) and incubated with an Fc-blocking antibody (CD16/CD32, clone 2.4G2, BD Biosciences) prior to extracellular staining. Extracellular staining was carried out at room temperature (RT) for 20 min, samples were fixed in 1% paraformaldehyde (PFA) and stored overnight at 4 °C until samples were acquired. For intracellular staining of IFN-γ, the Foxp3/Transcription Factor Staining Buffer Set (eBioscience™) was used following the manufacturer’s instructions. For YAC-1/NK co-culture experiments, cells were stained with Ethidium Homodimer (EthD-1, Invitrogen) to differentiate live/dead cells prior to flow cytometric analysis and were acquired on the same day. Samples were acquired on a BD LSR Fortessa or BD FACS-Celesta flow cytometer. Data were analyzed using FlowJo software (v10.8.1).

### Antibodies for flow cytometric analysis

Anti-CD45 (clone 30-F1), anti-CD3ε (clone 145-2C11), anti-CD4 (clone GK1.5), anti-CD8a (clone 53–6.7), anti-CD49b (clone DX5), anti NKR-P1C (clone PK136), anti-CD25 (clone PC61), anti-TIGIT (clone 1G9), anti-CD11b (clone M1/70), anti-Ly6C/Ly6G (clone R86-8C5), anti-Ly6C (clone Al-21), anti-F4/80 (clone T45-2342), CD11c (clone HL3), anti-MHC-II (I-A/I-E, clone M5/114), anti-CD86 (clone GL1), anti-CD40 (clone 3/23) were acquired from BD Biosciences. Anti-DNAM-1 (clone TX42.1), anti-CD31 (clone 390), and anti-IFN-γ (XMG1.2) were acquired from BioLegend.

### Immunohistochemistry (IHC)

IHC was performed on 5 μm sections of formalin-fixed paraffin-embedded tumour tissue as previously described^54^. Briefly, anti-FGL2 (LSBio, clone LS-C293889) antibody was diluted 1:500 and incubated with slides overnight at 4 °C, followed by anti-rabbit horseradish peroxidase–conjugated secondary antibody (Vector Laboratories) for 1 h at RT. Images were acquired using a Zeiss AxioScan Z1 (20X objective).

### Single-cell RNA sequencing (scRNA-seq)

*scRNA-seq* datasets (ovarian cancer^39,40^, melanoma^38^ , lung^39,41^, breast^39,42^, and colorectal^39,43^) were previously compiled and processed^83^. Analysis scripts are available at https://github.com/dpcook/emp_programs/blob/main/tumour_preprocessing.Rmd.

### *NK cell *in vitro* assays*

#### Degranulation/intracellular IFN-y

Flat-bottomed 96 well plates (Thermo Fisher Scientific) were coated with 5 μg of α-NKR-P1C (clone PK136, BD Biosciences) in 100μL PBS overnight. The following day plates were washed 3 × and 1 × 10^6^ splenocytes per well were stimulated for 5 h in RPMI + 5% FBS in the presence of 1:1000 Golgi Stop (BD BD Biosciences), 1000 IU/mL of recombinant murine IL-2 (BioLegend), and α-CD107a-AF647 (0.025 μg/well). Some cells were stimulated as above with the addition of 400 nM Phorbol myristate acetate (PMA, Sigma-Aldrich) and 3.35 μM ionomycin (Sigma-Aldrich).

#### Cytotoxicity

NK cells were isolated from splenocytes using EasySep™ Mouse NK Cell Isolation Kit (StemCell Technologies) according to the manufacturer’s instructions. 5 × 10^3^ YAC-1 target (T) cells were stained with CP450 (eBioscience) and cultured with isolated NK cells (Effector, E) at indicated ratios for 4 h in complete NK media. Cells were then stained with EthD-1 prior to flow cytometric analysis.

### Statistical analysis

All graphs were prepared in Prism 9.0 (GraphPad Software Inc.) or using R (ggplot2, pheatmap, Seurat). All statistical analyses were performed with Prism 9.0. Student’s *t* test was used to compare two groups or one-way analysis of variance (ANOVA) followed by Tukey’s multiple comparisons test to compare more than two groups. Survival data were depicted in Kaplan–Meier survival plots and statistical significance calculated by Log-rank (Mantel-Cox) tests. Results were considered statistically significant at *p* < 0.05 (*p < 0.05; **p < 0.01; ***p < 0.001; ****p < 0.0001). Data are presented as the means ± SD.

### Ethics approval and consent to participate

Human ascites supernatants were acquired from the Ottawa Ovarian Cancer Tissue Bank (Ottawa, Canada) with Research Ethics Board approval (Protocol 1999540-01H) and informed consent. Animal experiments were carried out using protocols approved by the Animal Care Committee at the University of Ottawa and conforming to the standards defined by the Canadian Council on Animal Care.

### Supplementary Information

Supplementary Information 1.

Supplementary Figures.

Supplementary Information 2.

Supplementary Table S1.

## Data Availability

*scRNA-seq* datasets were previously compiled and processed^37^. Analysis scripts are available at https://github.com/dpcook/emp_programs/blob/main/tumour_preprocessing.Rmd. All other data is available within the article and its supplementary materials.
