# Supplementary material for: FGL2 promotes tumour growth and attenuates infiltration of activated immune cells in melanoma and ovarian cancer models
Source: Sci Rep. 2024 Jan 8;14:787. doi: 10.1038/s41598-024-51217-1 (PMC10774293; doi:10.1038/s41598-024-51217-1)
Supplement: Supplementary file 1 — Supplementary Information 1. [file 41598_2024_51217_MOESM1_ESM.pdf]

Abnova (6D9)

Ladder

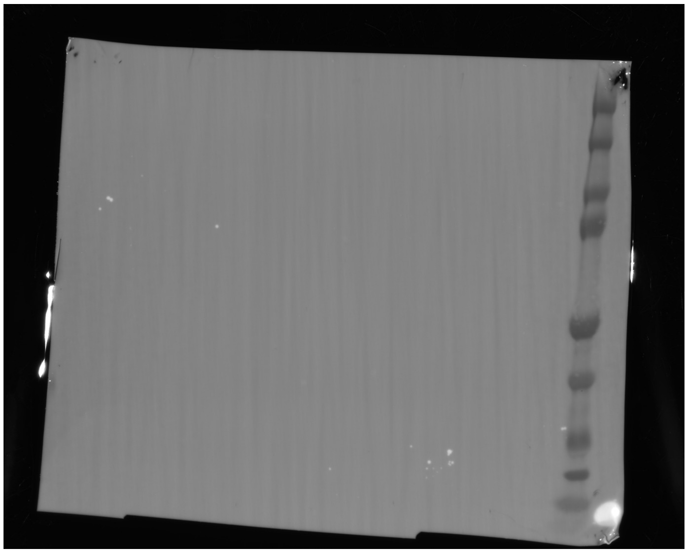

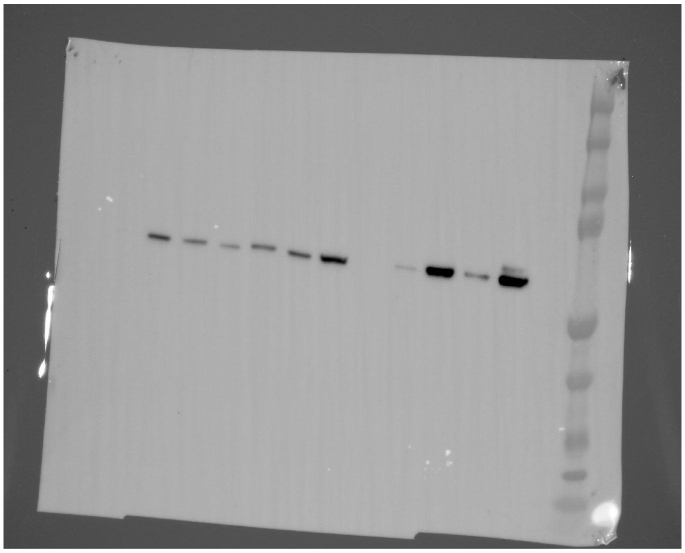

75KDa
FGL2
50KDa

FGL2 - Abnova

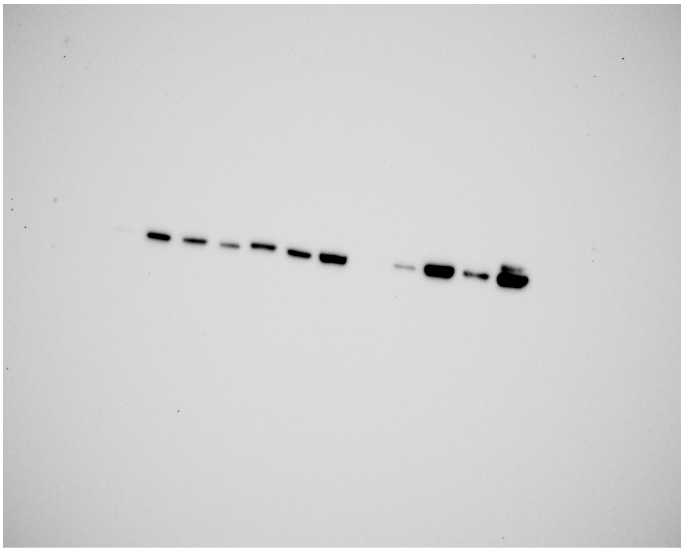

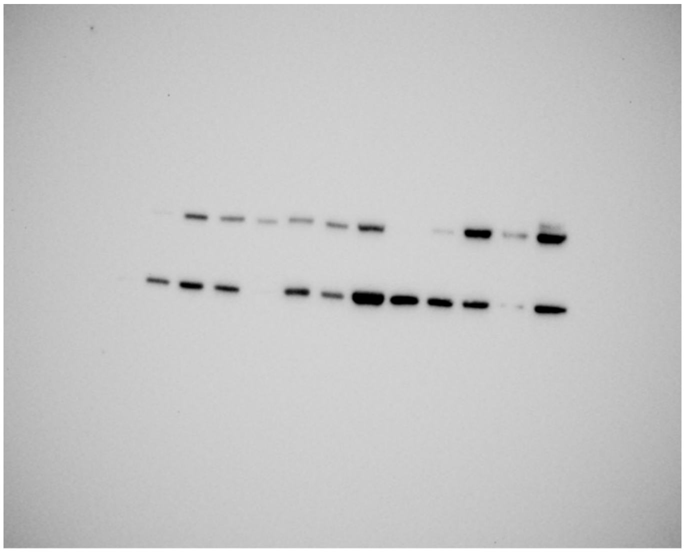

FGL2
B-Actin

Polyclonal-aFGL2 (GL)

Ladder

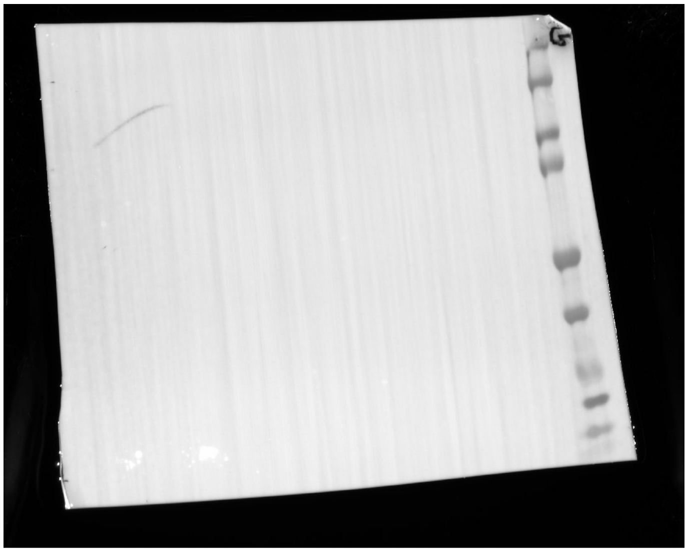

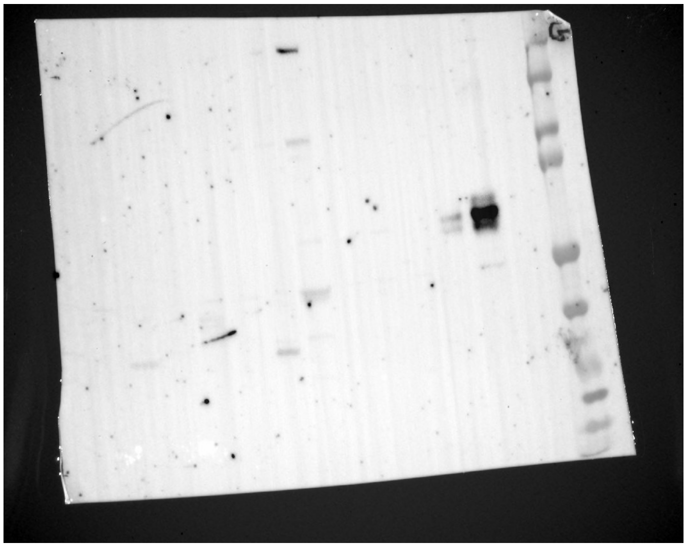

75KDa
FGL2
50KDa

FGL2 - Abnova

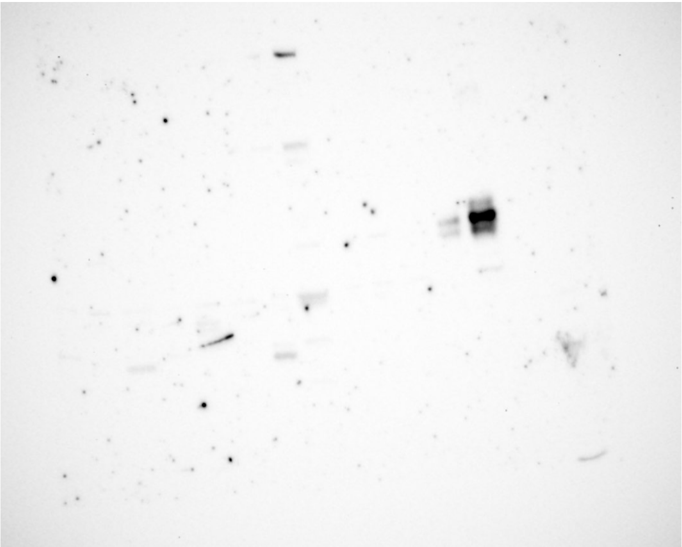

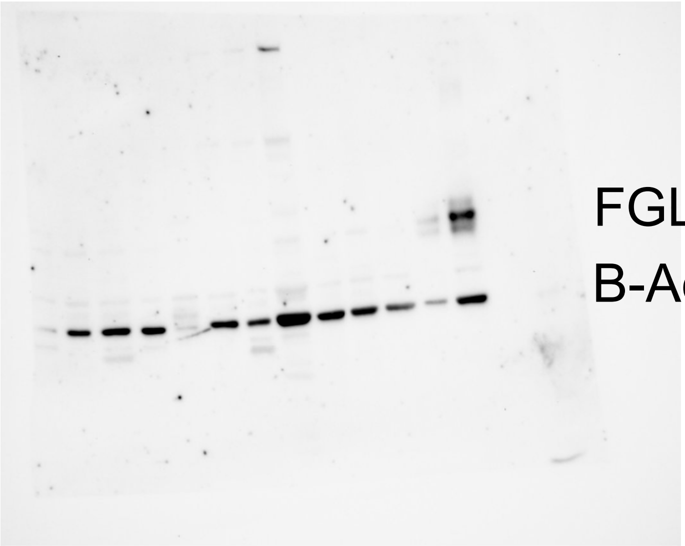

FGL2
B-Actin
