# Supplementary material for: FGL2 promotes tumour growth and attenuates infiltration of activated immune cells in melanoma and ovarian cancer models
Source: Sci Rep. 2024 Jan 8;14:787. doi: 10.1038/s41598-024-51217-1 (PMC10774293; doi:10.1038/s41598-024-51217-1)
Supplement: Supplementary file 2 — Supplementary Figures. [file 41598_2024_51217_MOESM2_ESM.pdf]

Supplemental Figures

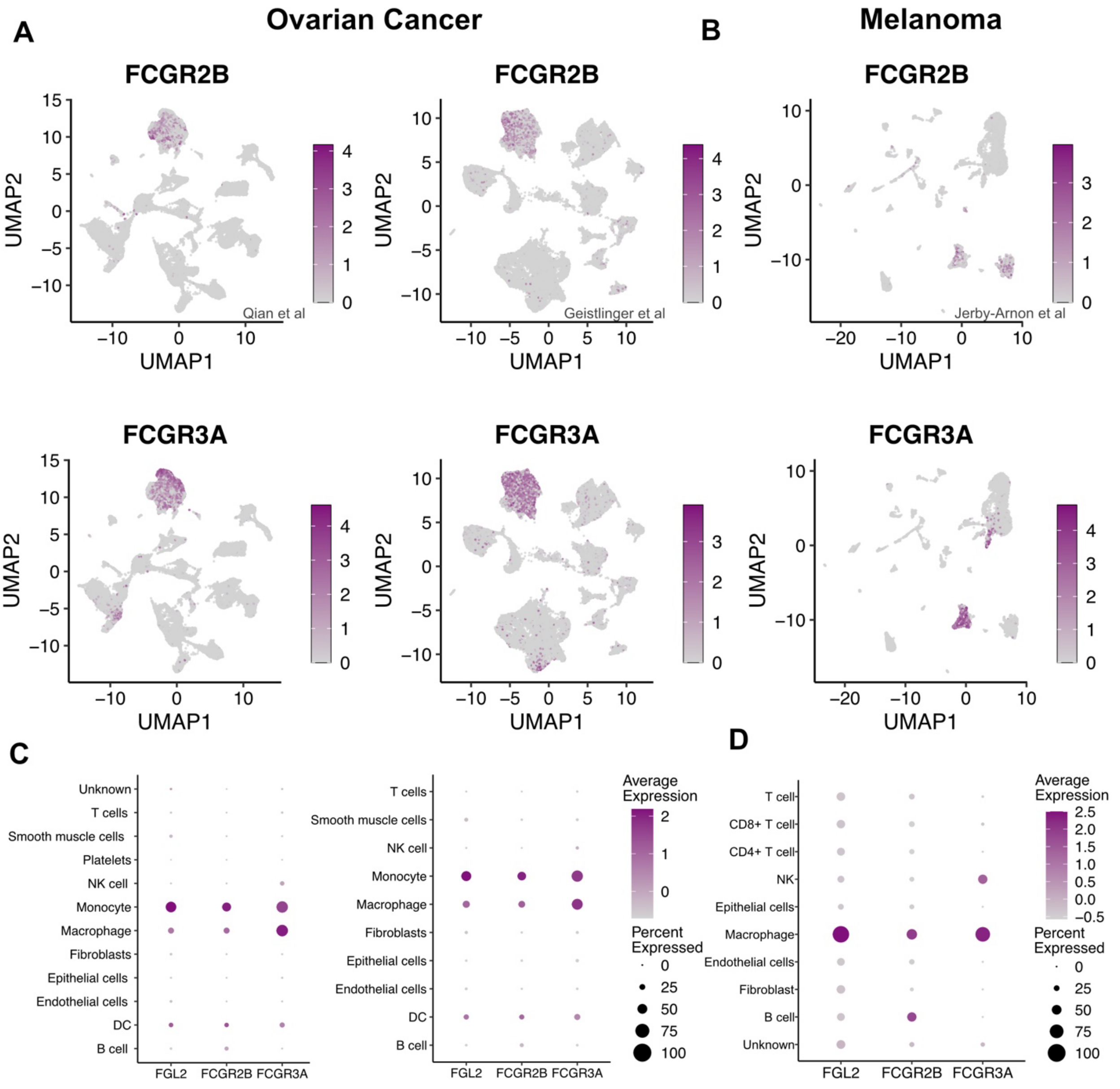

Figure S1. Single-cell RNA sequencing data indicates that FGL2 receptors FcγRIIB and FcγRIII are primarily expressed in macrophages of human ovarian cancer and melanoma. UMAP embedding of scRNA-seq data from human **(A)** ovarian cancer [Left: Qian et al.[39] (n= 5) , Right: Geistlinger et al.[40] (n=5)] datasets and **(B)** human melanoma dataset[38](n= 33) depict heatmaps displaying the level of expression of FcγRIIB (top) and FcγRIII (bottom) in each cell type cluster. Average expression values of FGL2, FcγRIIB, and FcγRIII in each cell type cluster in the same datasets of **(C)** ovarian cancer and **(D)** melanoma.

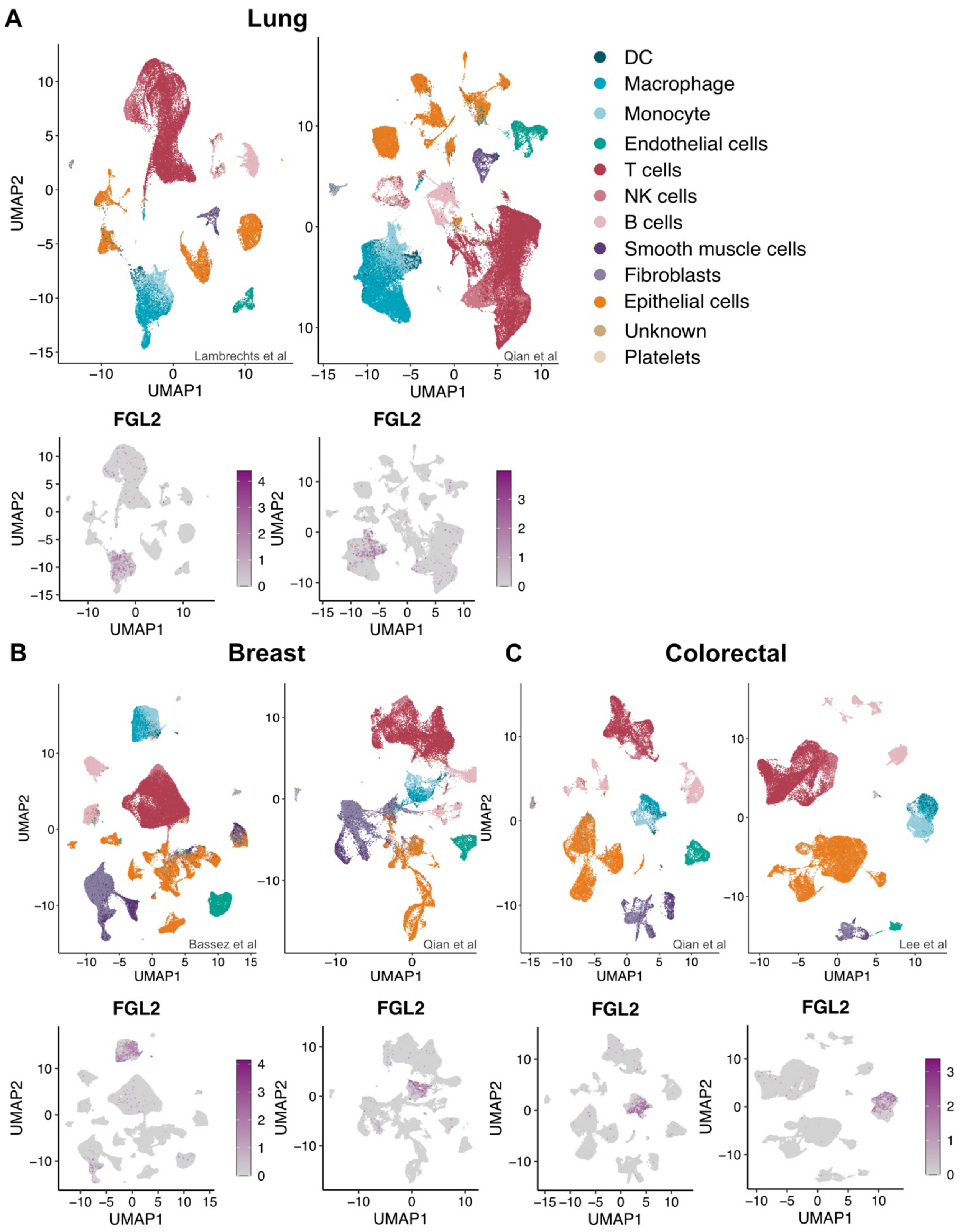

A
Lung
B
Breast
C
Colorectal
DC
Macrophage
Monocyte
Endothelial cells
T cells
NK cells
B cells
Smooth muscle cells
Fibroblasts
Epithelial cells
Unknown
Platelets
Lambrechts et al
Qian et al
Bassez et al
Lee et al
FGL2
UMAP1
UMAP2

Figure S2. Single-cell RNA sequencing data indicates FGL2 is primarily expressed in macrophages of human lung, breast and colorectal carcinomas. UMAP embedding of scRNA-seq data from human **(A)** lung [Left: Lambrechts et al.[41](n=9), Right: Qian et al.[39](n=24) **(B)** breast [Left: Bassez et al.[42](n=62), Right: Qian et al.[39](n=10)] **(C)** colorectal [Left: Qian et al.[39] (n=11), Right: Lee et al.[43](n=25)] datasets depicting cell clusters with accompanying heatmap displaying the level of expression of FGL2 in each cell type cluster.

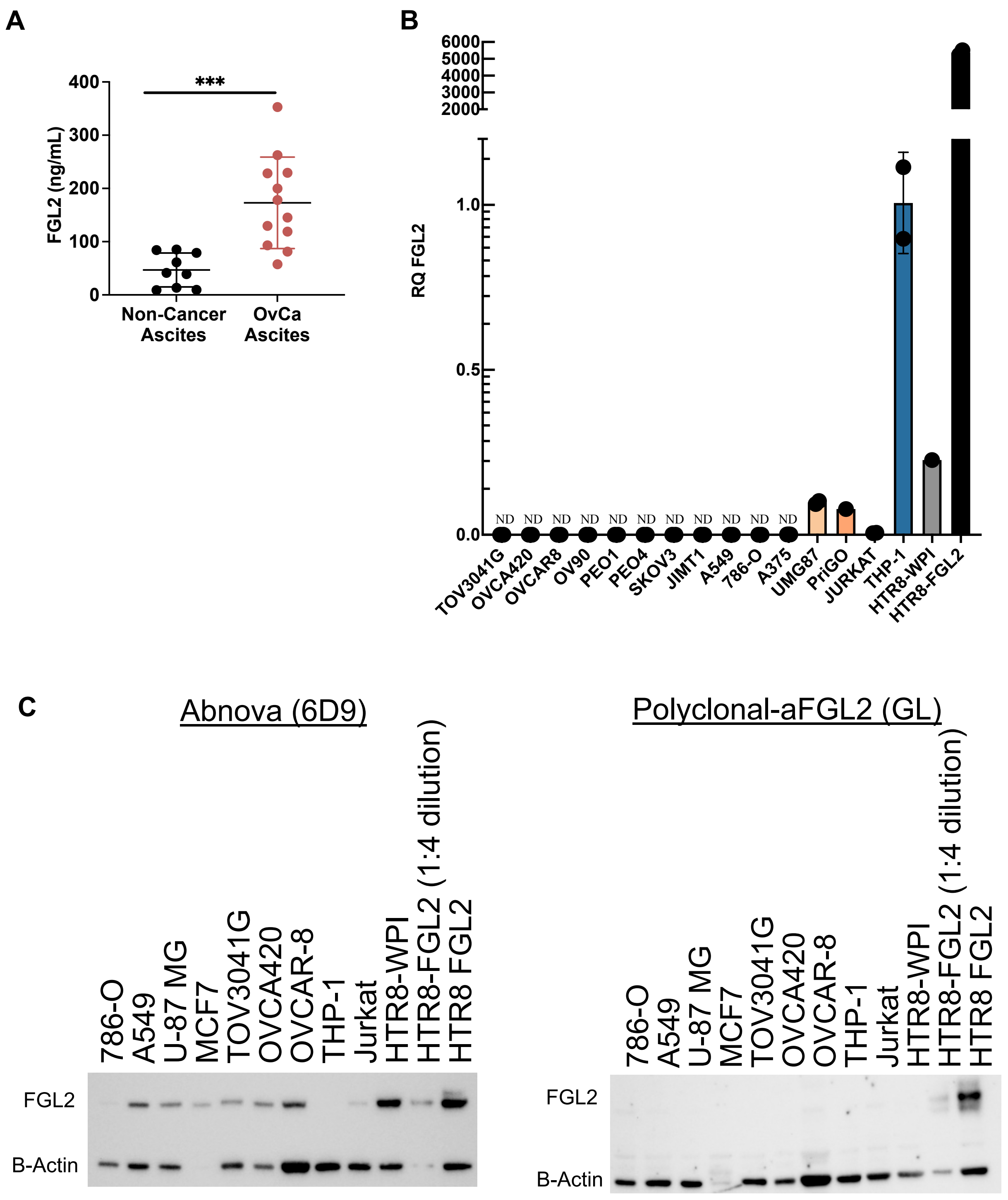

A
B
C
FGL2 (ng/mL)
***
Non-Cancer Ascites
OvCa Ascites
RQ FGL2
ND
TOV3041G
OVCA420
OVCAR8
OV90
PEO1
PEO4
SKOV3
JIMT1
A549
786-O
A375
UMG87
PriGO
JURKAT
THP-1
HTR8-WPI
HTR8-FGL2
Abnova (6D9)
Polyclonal-aFGL2 (GL)
786-O
A549
U-87 MG
MCF7
TOV3041G
OVCA420
OVCAR-8
THP-1
Jurkat
HTR8-WPI
HTR8-FGL2 (1:4 dilution)
HTR8 FGL2
FGL2
B-Actin

Figure S3. FGL2 expression is not detected in epithelial cancer cell lines but FGL2 protein is detected at elevated levels in ovarian cancer ascites. **(A)** FGL2 concentrations were determined by ELISA in the ascites fluid from ovarian cancer patients (n=12) and non-cancer ascites fluid (n=7). Significance determined by Student's t test. ***$p \leq 0.001$. **(B)** *FGL2* transcripts were detected at low levels in U-87 MG and PriGO glioblastoma cell lines as well as the immune cell derived Jurkat (T cells) and THP-1 (monocytes) but undetectable in ovarian cancer cell lines (TOV3041G, OVCA420, OVCAR8, PEO1, PEO4, SKOV3, OV90), as well as melanoma (A375), lung (A549), renal (786-O) and breast (JIMT1) cell lines. FGL2 was also detected in our control cell line (placental, HTR-8-WPI) with elevated levels when FGL2 was overexpressed (HTR-8-FGL2). n=3-4/cell line. **(C)** Western blot detection of FGL2 on identical protein samples run in parallel and detected by either Abnova (6D9) or a polyclonal in-house generated antibody. Beta-actin serves as a loading control.

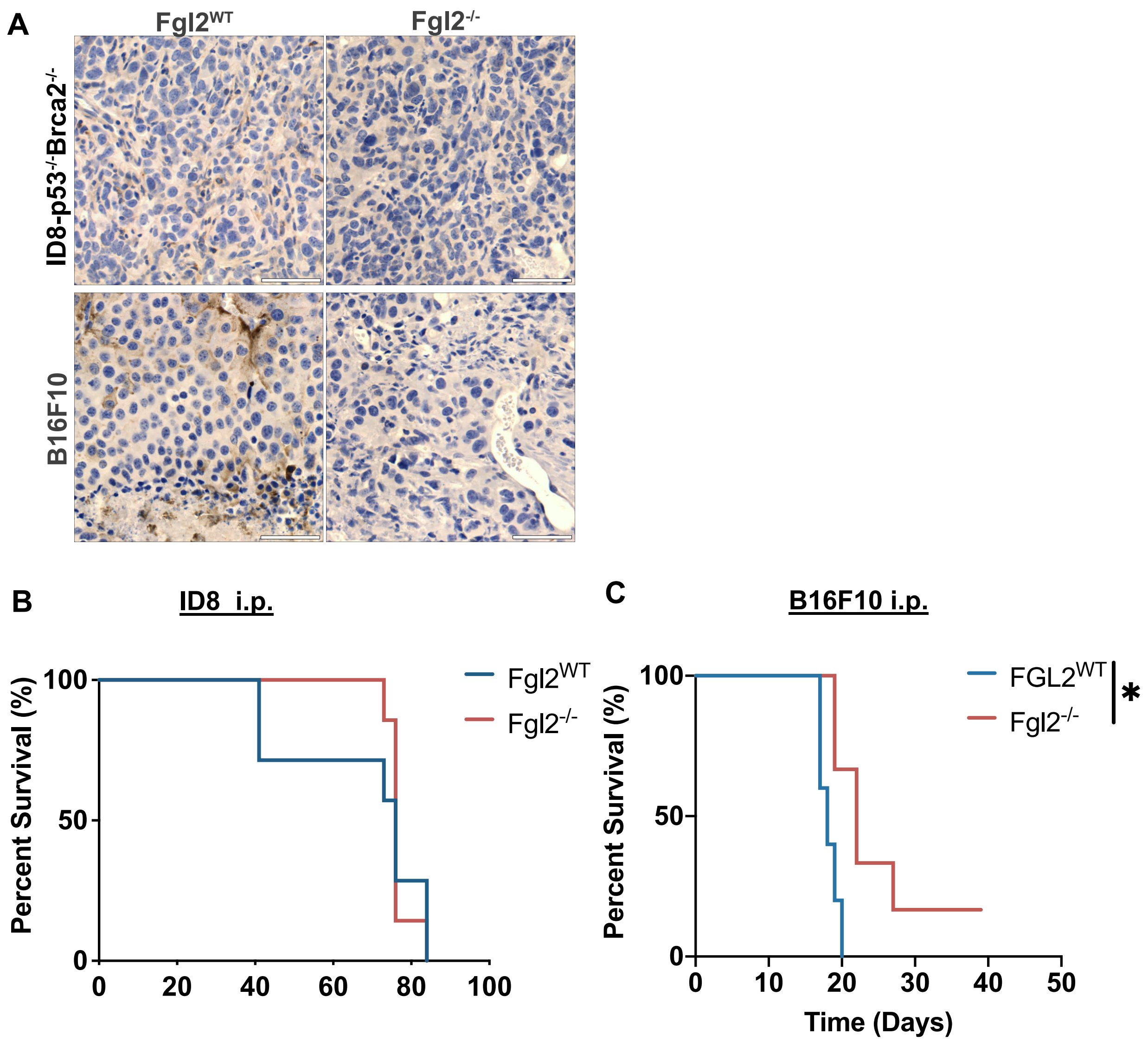

Figure S4. Absence of FGL2 slows tumour progression and prolongs survival of B16F10 melanoma models. (A) IHC detection of FGL2 in *Fgl2*^{WT} mice. Sections from tumours from Figure 2A and 2D [i.p. injection of $5x10^6$ ID8-*p53*$^{-/-}$*Brca2*$^{-/-}$ cells (n=6/group) or s.c injection of $2x10^5$ B16F10cells (n=6-8/group)] were counterstained with hematoxylin (blue) and positive cells (brown) were stained with DAB. Images are representative of each tumour model. Scale bars, = 50 μm. **(B)** *Fgl2*^{WT} or *Fgl2*$^{-/-}$ mice received an i.p. injection of ID8 cells (n=6/group) and were monitored for survival until humane endpoint. Significance was determined by Log-rank test. **(C)** *Fgl2*^{WT} and *Fgl2*$^{-/-}$ mice received an i.p. injection of 25 x 10^4 B16F10 cells (n=6/group) and were monitored for survival until humane endpoint. Significance was determined by Log-rank test, *$p \leq 0.05$.

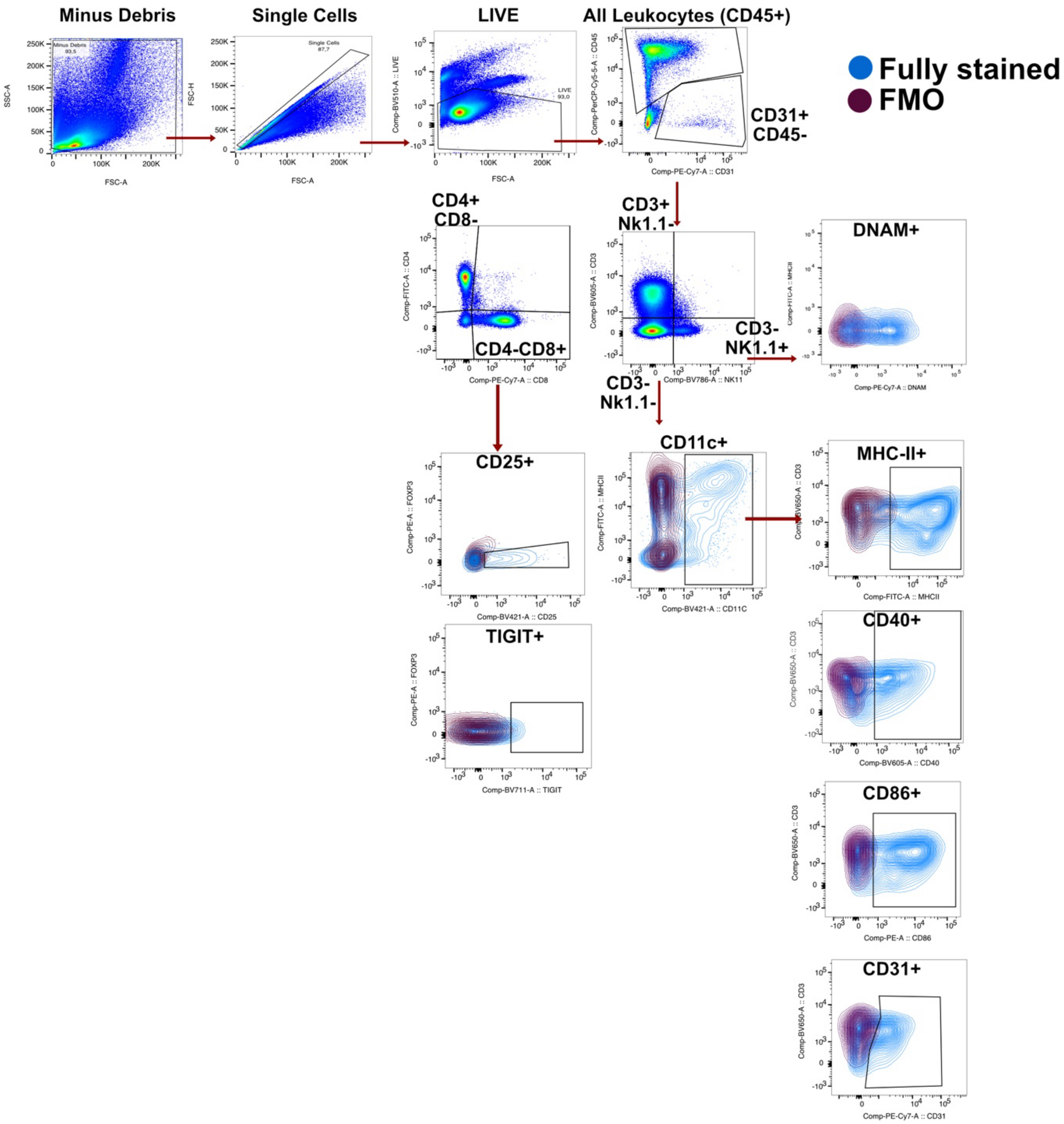

Figure S5. Gating strategy for analysis of flow cytometry data. Gating strategy used to analyze the flow cytometry data from murine tumours and spleens are as follows: cell debris exclusion (Minus Debris), singlet, live/dead exclusion (LIVE), CD45+ (All Leukocytes), NK Cells (CD3- NK1.1+), T cells (CD3+ NK1.1-), CD4+ T cells (CD4+ CD8-), CD8+ T cells (CD4-CD8+), and dendritic cells (CD3- CD11c+). CD25, TIGIT, DNAM-1, MHC-II, CD86, CD40 and CD31 were further used to phenotype immune cell populations. Contour plots show fluorescence minus one (FMOs) for each analyzed marker.

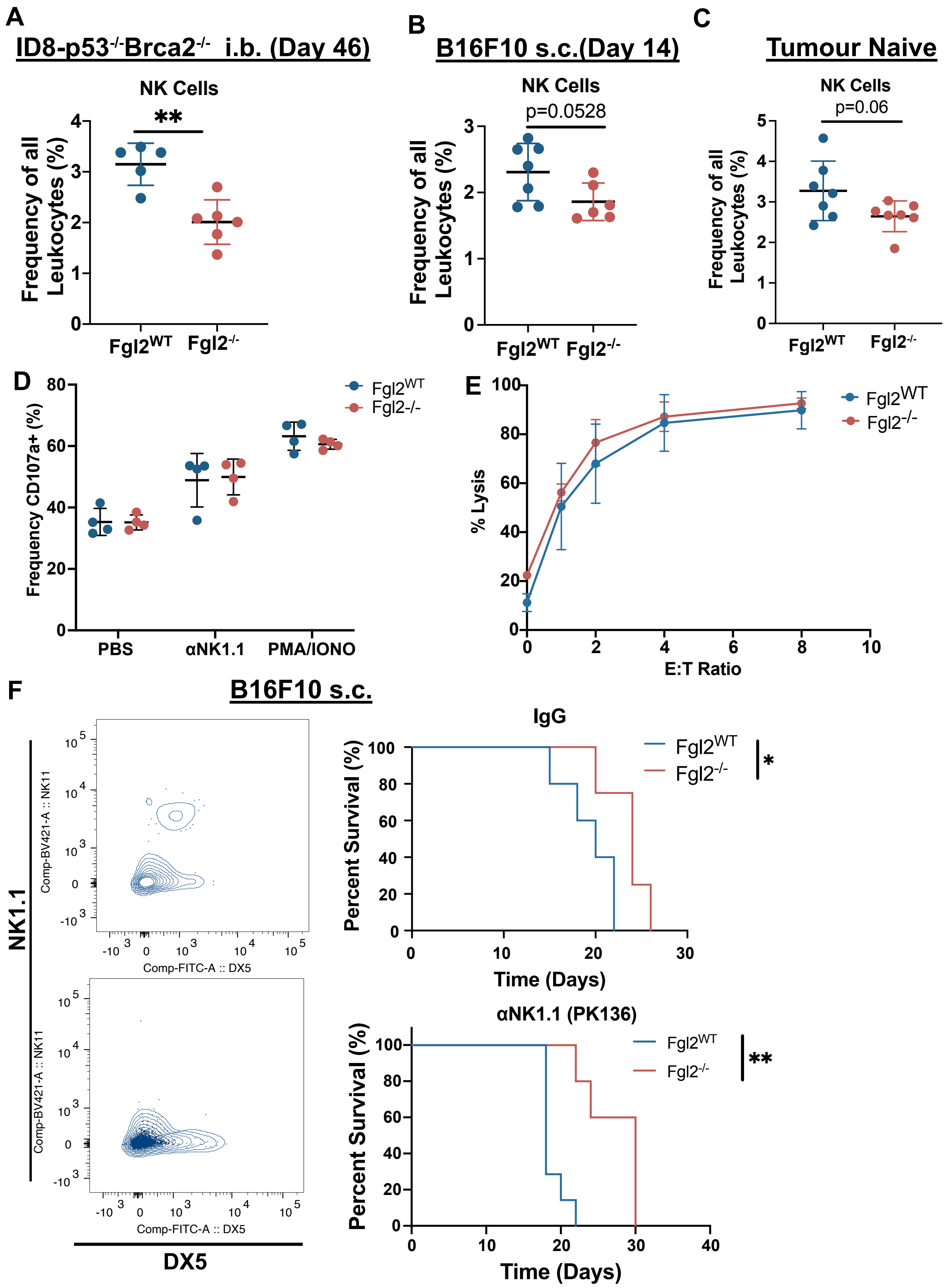

A
ID8-p53-/-Brca2-/- i.b. (Day 46)
NK Cells
**
Frequency of all Leukocytes (%)
Fgl2WT
Fgl2-/-
B
B16F10 s.c.(Day 14)
NK Cells
p=0.0528
Frequency of all Leukocytes (%)
Fgl2WT
Fgl2-/-
C
Tumour Naive
NK Cells
p=0.06
Frequency of all Leukocytes (%)
Fgl2WT
Fgl2-/-
D
Frequency CD107a+ (%)
Fgl2WT
Fgl2-/-
PBS
αNK1.1
PMA/IONO
E
% Lysis
Fgl2WT
Fgl2-/-
E:T Ratio
F
B16F10 s.c.
NK1.1
Comp-BV421-A :: NK11
Comp-FITC-A :: DX5
DX5
IgG
Percent Survival (%)
Time (Days)
Fgl2WT
Fgl2-/-
*
αNK1.1 (PK136)
Percent Survival (%)
Time (Days)
Fgl2WT
Fgl2-/-
**

Figure S6. NK cells do not contribute to prolonged survival in *Fgl2*$^{-/-}$ mice. *Fgl2*^{WT} and *Fgl2*$^{-/-}$ mice received bilateral i.b. injections of ID8-*p53*$^{-/-}$*Brca2*$^{-/-}$ cells (n=5/group) or s.c. injections of B16F10 cells (n=7/group). Spleens were collected on day 46 **(A)** or day 14 **(B)** respectively, as well as from **(C)** tumour-naïve mice (n=3-7 mice/group) and assessed by flow cytometry. Gating strategy: Minus Debris/Singlets/Live Cells/CD45+, NK Cells (DX5+CD3-). **(D)** NK cells from *Fgl2*^{WT} and *Fgl2*$^{-/-}$ mice were assessed for degranulation (CD107+[66]) by plate-bound activation with anti-NK1.1, non-specific activation by PMA/Ionomycin, or with PBS as an unactivated control. Gating strategy: Minus Debris/Singlets/Live Cells/CD45+/NK Cells (NK1.1+CD3-) and CD107+ NK cells. **(E)** NK cells from *Fgl2*^{WT} and *Fgl2*$^{-/-}$ mice were assessed for their ability to induce target cell lysis at different ratios of effector (E): target (T) cell (YAC-1) ratios. Gating strategy: Minus Debris/Singlets/NK Cells (CP450+)/lysed cells (PI+). Significance determined by Student's t test. **(F)** *Fgl2*^{WT} and *Fgl2*$^{-/-}$ mice (n=6mice/group) were treated 200μg/mouse IgG control antibody or anti-NK1.1 (PK136) to deplete NK cells one and two days before tumour injections (B16F10 2x10^5 cells s.c.) followed by weekly injection of depletion antibodies. Mice were assessed for survival and spleens were collected at endpoint to confirm depletion of NK cells. Gating strategy: Minus Debris/Singlets/Live Cells/CD45+, NK Cells (NK1.1+DX5+CD3-). Significance determined by Log-Rank test, $*p \leq 0.05$, $**p \leq 0.01$.

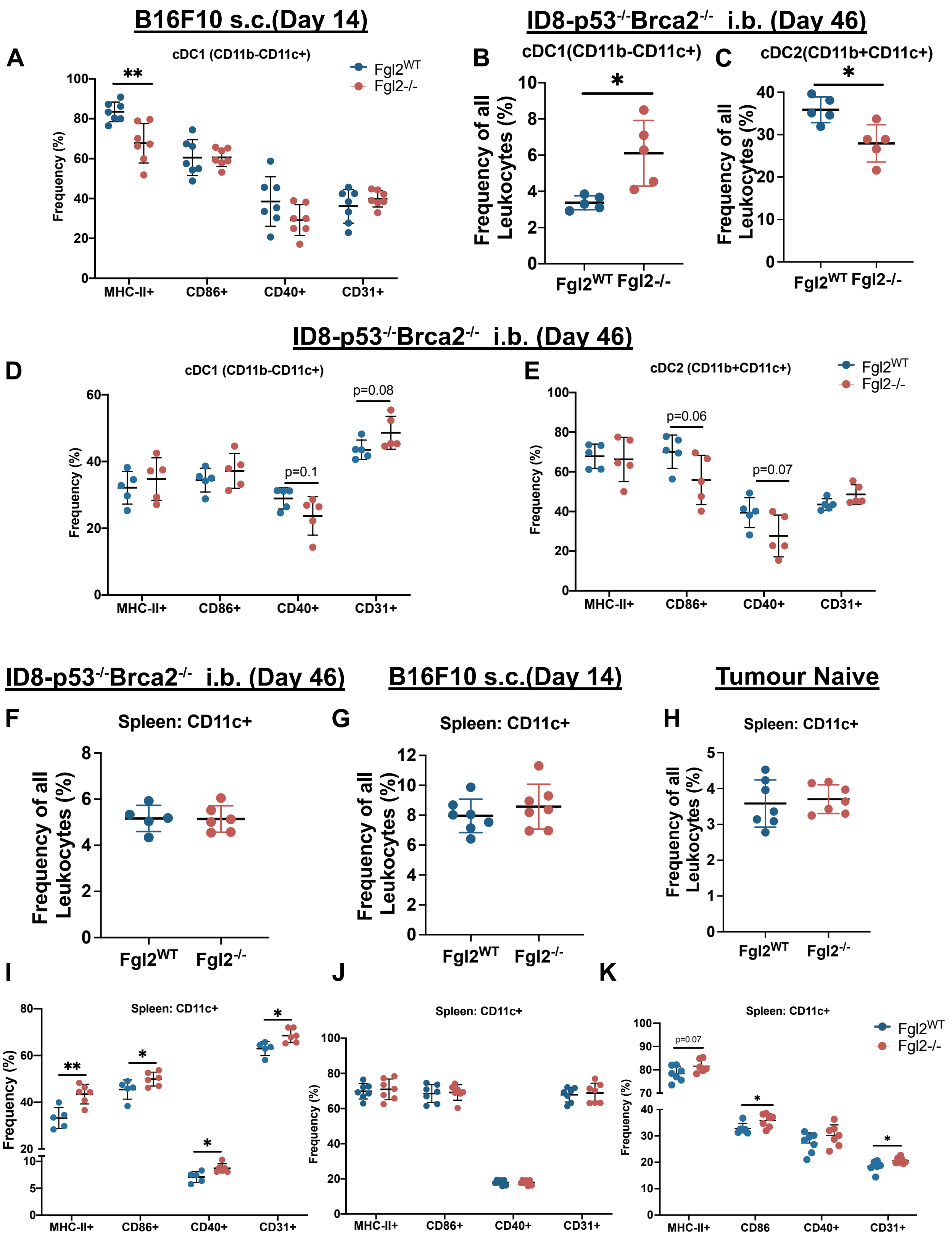
B16F10 s.c.(Day 14)
ID8-p53-/-Brca2-/- i.b. (Day 46)
A
cDC1 (CD11b-CD11c+)
Fgl2WT
Fgl2-/-
Frequency (%)
MHC-II+
CD86+
CD40+
CD31+
B
cDC1(CD11b-CD11c+)
Frequency of all Leukocytes (%)
Fgl2WT Fgl2-/-
C
cDC2(CD11b+CD11c+)
ID8-p53-/-Brca2-/- i.b. (Day 46)
D
cDC1 (CD11b-CD11c+)
p=0.1
p=0.08
E
cDC2 (CD11b+CD11c+)
p=0.06
p=0.07
ID8-p53-/-Brca2-/- i.b. (Day 46)
B16F10 s.c.(Day 14)
Tumour Naive
F
Spleen: CD11c+
G
Spleen: CD11c+
H
Spleen: CD11c+
I
Spleen: CD11c+
J
Spleen: CD11c+
K
Spleen: CD11c+
p=0.07
CD86

Figure S7: The absence of FGL2 results in activated/mature splenic DCs. *Fgl2*^{WT} and *Fgl2*$^{-/-}$ mice received bilateral i.b. injections of ID8-*p53*$^{-/-}$*Brca2*$^{-/-}$ cells (n=5/group) or s.c. injections of B16F10 cells (n=7/group). Tumours **(A-E)** and spleens **(F-K)** were collected on day 14**(A, G, J)** or day 46 **(B-F, I)**, as well as from **(H,K)** tumour-naïve mice (n=3-7 mice/group). Frequency of cDC1s **(A,B),** cDC2s **(C),** and DCs (**F-H**) with frequency of MHC-II+, CD86+, CD31+, and CD40+ DCs of tumour-bearing mice and tumour-naïve mice were assessed by flow cytometry. Gating strategy: Minus Debris/Singlets/Live Cells/CD45+, followed by: cDC1s (CD3-CD11b-CD11c+), cDC2s (CD3-CD11b+CD11c+), DCs (CD3-CD11c+) and MHC-II, CD86, CD40, and CD31 expression. Significance determined by Student's t test; *$p \leq 0.05$, **$p \leq 0.01$.

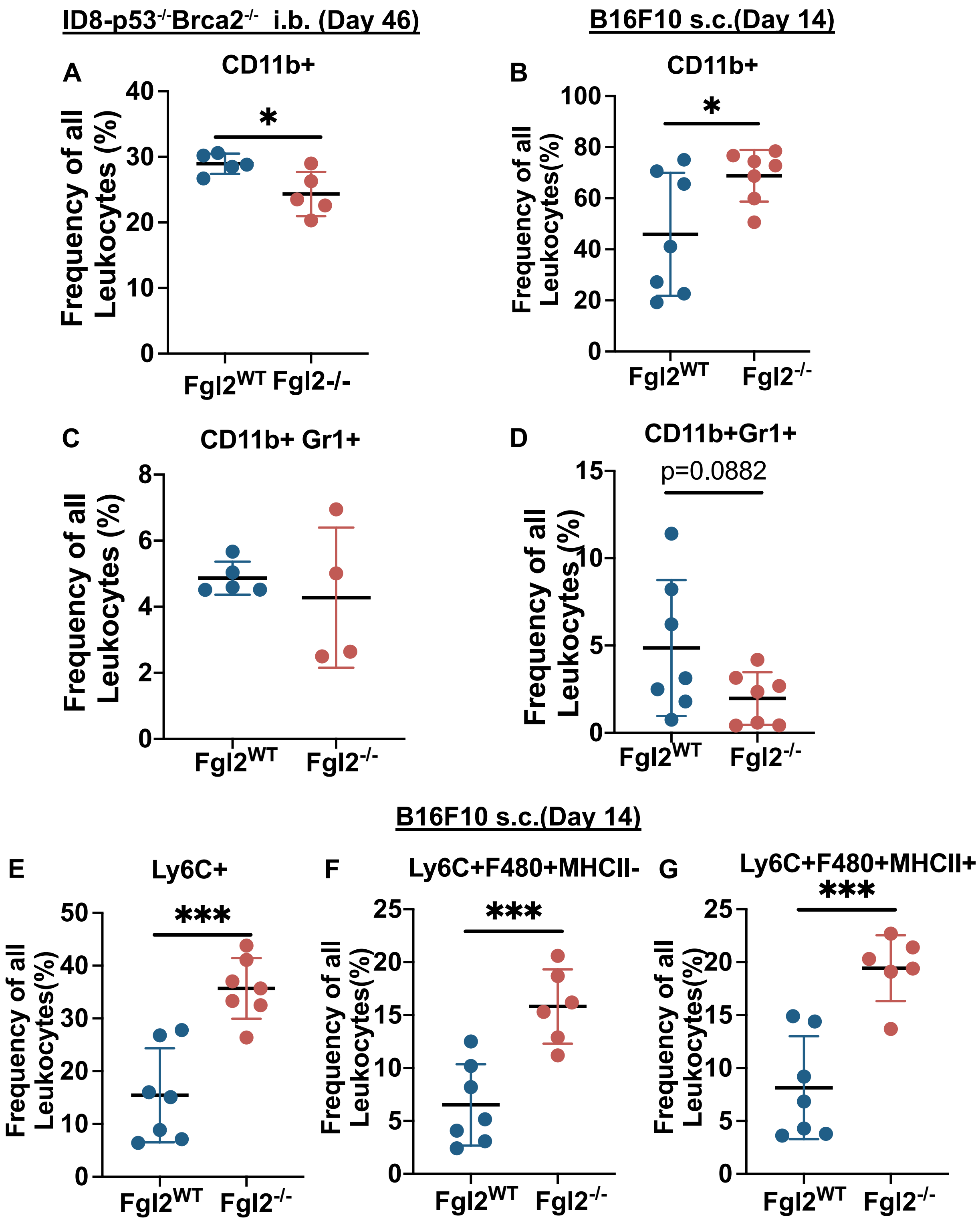
ID8-p53-/-Brca2-/- i.b. (Day 46)
B16F10 s.c.(Day 14)
A
CD11b+
Frequency of all Leukocytes (%)
*
Fgl2WT Fgl2-/-
B
CD11b+
Frequency of all Leukocytes(%)
*
Fgl2WT Fgl2-/-
C
CD11b+ Gr1+
Frequency of all Leukocytes (%)
Fgl2WT Fgl2-/-
D
CD11b+Gr1+
p=0.0882
Frequency of all Leukocytes (%)
Fgl2WT Fgl2-/-
B16F10 s.c.(Day 14)
E
Ly6C+
***
Frequency of all Leukocytes(%)
Fgl2WT Fgl2-/-
F
Ly6C+F480+MHCII-
***
Frequency of all Leukocytes(%)
Fgl2WT Fgl2-/-
G
Ly6C+F480+MHCII+
***
Frequency of all Leukocytes(%)
Fgl2WT Fgl2-/-

Figure S8: The absence of FGL2 results in increased myeloid cells in B16F10 tumours. *Fgl2*^{WT} and *Fgl2*$^{-/-}$ mice received bilateral i.b. injections of ID8-*p53*$^{-/-}$*Brca2*$^{-/-}$ cells (n=5/group) or s.c. injections of B16F10 cells (n=7/group). Tumours were collected on day 46 **(A, C)** or day 14 **(B, D-G)** respectively, and the frequency of myeloid cell populations was assessed by flow cytometry. Gating strategy: Minus Debris/Singlets/Live Cells/CD45+, followed by: **A,B**) myeloid cells (CD3-CD11c-CD11b+), **C,D**) CD11b+Gr1High (CD3-CD11c-) populations, and **E)** Ly6C+ (CD3-CD11c-), **F)** monocytes (F480+MHCII-) and **G)** macrophages (F480+MHCII+). Significance determined by Student's t test; *p≤0.05, **p≤ 0.01, ****p<0.001.

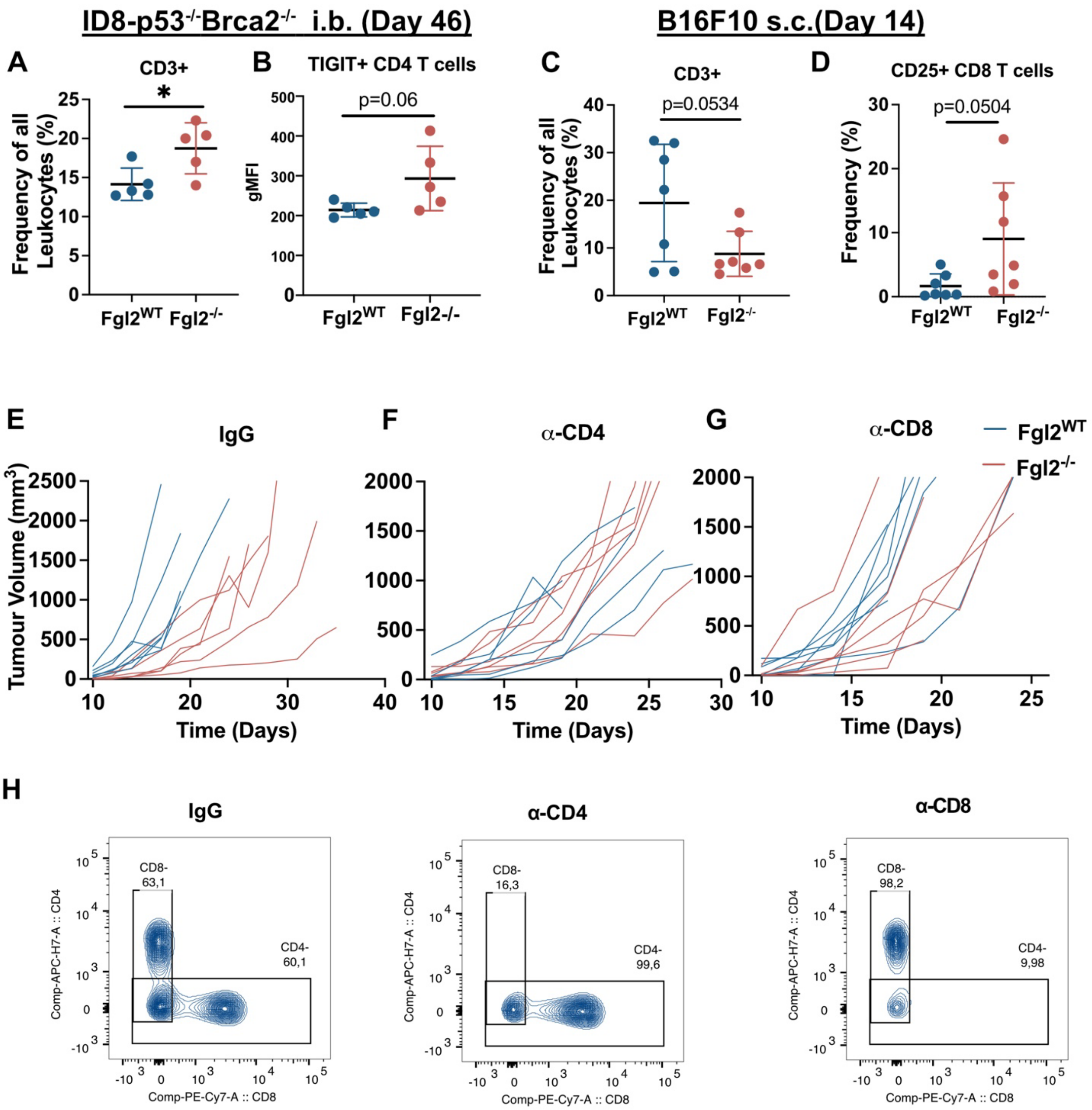
ID8-p53-/-Brca2-/- i.b. (Day 46)
B16F10 s.c.(Day 14)
A
CD3+
Frequency of all Leukocytes (%)
Fgl2WT Fgl2-/-
B
TIGIT+ CD4 T cells
p=0.06
gMFI
Fgl2WT Fgl2-/-
C
CD3+
p=0.0534
Frequency of all Leukocytes (%)
Fgl2WT Fgl2-/-
D
CD25+ CD8 T cells
p=0.0504
Frequency (%)
Fgl2WT Fgl2-/-
E
IgG
F
α-CD4
G
α-CD8
Fgl2WT
Fgl2-/-
Tumour Volume (mm3)
Time (Days)
H
IgG
α-CD4
α-CD8
CD8-
63,1
CD4-
60,1
CD8-
16,3
CD4-
99,6
CD8-
98,2
CD4-
9,98
Comp-APC-H7-A :: CD4
Comp-PE-Cy7-A :: CD8

Figure S9: The absence of FGL2 in the tumour microenvironment increases T cells in ID8-*p53*$^{-/-}$*Brca2*$^{-/-}$ tumours and activated T cells in B16F10 tumours. *Fgl2*^{WT} and *Fgl2*$^{-/-}$ mice received bilateral i.b. injection of $0.15x10^6$ ID8-*p53*$^{-/-}$*Brca2*$^{-/-}$ cells (n=5/group) or s.c. injection of $2x10^5$ B16F10 cells (n=7/group). Tumours were collected on (**A, B**) day 46 or (**C, D**) day 14 respectively. Total frequency of T cells was assessed by flow cytometry in **(A)** ID8-*p53*$^{-/-}$*Brca2*$^{-/-}$ tumours and **(C)** B16F10 tumours. **(B)** TIGIT expression (gMFI) was assessed in CD4+ T cells in ID8-p53$^{-/-}$Brca2$^{-/-}$ tumours and **(D)** frequency of CD25+CD8+ T cells was assessed in B16F10 tumours in are more *Fgl2*$^{-/-}$ mice. Gating strategy: Minus Debris/Singlets/Live Cells/CD45+, T cells (NK1.1-CD3+) and CD4+/CD8+, and CD25+, TIGIT+ frequency/expression. Significance determined by Student's t test. *p≤ 0.05). *Fgl2*^{WT} and *Fgl2*$^{-/-}$ mice **(**n=6mice/group) were treated 500μg/mouse IgG control **(E)** antibody or **(F)** anti-CD4 or **(G)** 250μg/mouse anti-CD8 to deplete T cells one and two days before tumour injections (B16F10 $2x10^5$ cells s.c.) followed by twice weekly injections of 200μg/mouse of depletion antibodies. Tumour progression was measured approximately every two days using calipers to determine tumour volume ((WxWxL)/2) and is depicted by spider plots for each group. **(H)** Spleens were collected at endpoint to confirm depletion of T cells. Gating strategy: Minus Debris/Singlets/Live Cells/CD45+, CD4+ T cells (CD3+CD8-) or CD8+ (CD3+CD4-).

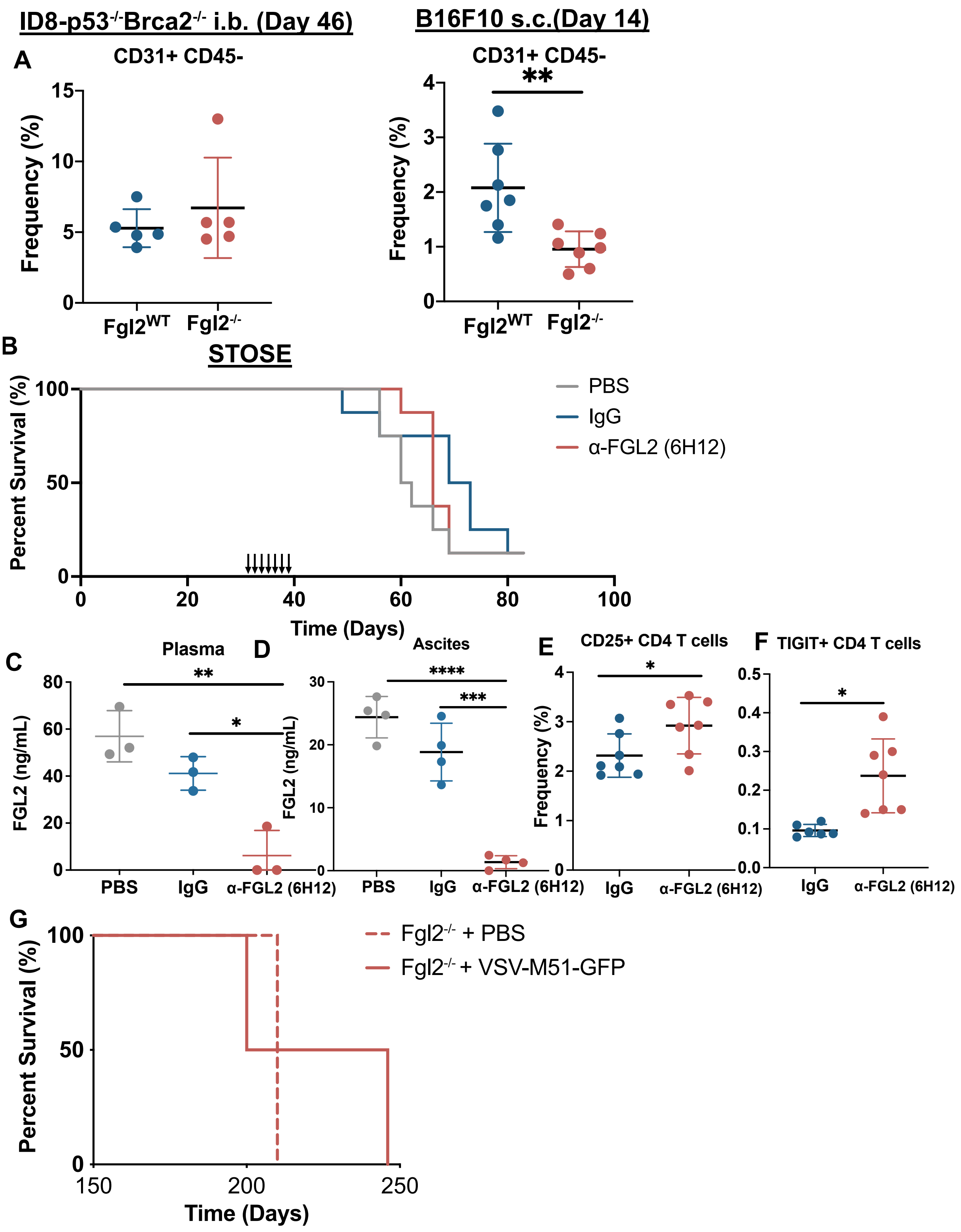
ID8-p53-/-Brca2-/- i.b. (Day 46)
B16F10 s.c.(Day 14)
A
CD31+ CD45-
Frequency (%)
Fgl2WT
Fgl2-/-
**
B
STOSE
Percent Survival (%)
Time (Days)
PBS
IgG
α-FGL2 (6H12)
C
Plasma
FGL2 (ng/mL)
D
Ascites
E
CD25+ CD4 T cells
F
TIGIT+ CD4 T cells
G
Fgl2-/- + PBS
Fgl2-/- + VSV-M51-GFP

Figure S10: α-FGL2 treatment of a STOSE model of ovarian cancer increases frequency of activated T cells. (A) *Fgl2*^{WT} and *Fgl2*$^{-/-}$ mice received bilateral i.b. injections of ID8-*p53*$^{-/-}$*Brca2*$^{-/-}$ cells (n=5/group) or s.c. injections of B16F10 cells (n=7/group) and the frequency of endothelial cells (CD31+ CD45-) in the tumours was assessed on day 46 (left panel) or day 14 (right panel) respectively. FVB/N mice bearing STOSE tumours (31 days post i.p. injection of $5x10^6$ STOSE cells) received 1 daily i.p. injection of PBS, IgG control, or α-FGL2 antibody (150μg/mouse) for 7 days. 24 hours after the final treatment, 7 mice/group were sacrificed, and spleen was analyzed by flow cytometry. (**B)** The remaining 8 mice/group were assessed for survival and significance was determined by Log-Rank test. Levels of FGL2 was determined by ELISA in the **(C)** in the plasma collected 24 hours following the final treatment or **(D)** ascites at endpoint. Frequencies of activated **(E)** CD25+ and **(F)** TIGIT+ CD4 T cells were assessed by flow cytometry in the spleen. Gating Strategy: Minus Debris/Singlets/Live Cells/CD45+, CD4+ T cells (NK1.1- CD3+CD4+), CD25+ or TIGIT+. Significance was determined by One-Way ANOVA with Tukey's post-test; *p≤0.05, **p≤ 0.01, ****p<0.0001. (**G)** Surviving mice from **Figure 5C** were re-challenged with $3x10^6$ *ID8-p53*$^{-/-}$*Brca2*$^{-/-}$ cells i.p. on day 150 and assessed for survival.
