# Supplementary material for: FGL2 promotes tumour growth and attenuates infiltration of activated immune cells in melanoma and ovarian cancer models
Source: Sci Rep. 2024 Jan 8;14:787. doi: 10.1038/s41598-024-51217-1 (PMC10774293; doi:10.1038/s41598-024-51217-1)
Supplement: Supplementary file 3 — Supplementary Information 2. [file 41598_2024_51217_MOESM3_ESM.docx]

**Supplemental Methods**

***Cell lines:*** Cell lines were maintained in indicated media: RPMI-10% FBS (786-O, OVCA420, THP-1, Jurkat, HTR8-WPI and HTR8-FGL2), DMEM-10% FBS (U87 MG, A549, MCF-7, OVCAR-8) or OSE media with 10% FBS (TOV3041G). Cells were collected near confluency (80-90%), washed twice with PBS, and cell pellets resuspended in RLT Plus Lysis Buffer (Qiagen) for RNA extraction or M-PER Lysis buffer for protein extraction (ThermoFisher). Placental cell line HTR-8/SVneo with overexpression of FGL2 acted as our positive control. Human FGL2 cDNA was purchased (Integrated DNA Technologies) and cloned into the pWPI vector (Addgene plasmid #12254). pWPI and pWPI-hFGL2 plasmids were packaged into a lentivirus, by transfecting HEK293 cells with purified DNA and packaging components. The HTR-8/SVneo cell lines were infected with both viruses (polybrene) and the successfully infected cells were isolated using fluorescence-activated cell sorting for GFP expression.

***RT-qPCR:*** RNA was extracted according to manufacturer’s protocol with the RNeasy Plus Mini Kit (Qiagen). cDNA was prepared with iScript Reverse Transcription Supermix (Bio-Rad), using 1μg of RNA. Relative gene expression was determined by qPCR SYBR Green Supermix (Bio-Rad) using the ABI 7500 FAST (Applied Biosystems) machine and its software. Gene expression was calculated as fold increase over THP-1 cells (monocytes) and normalized to the housekeeping gene *Ppia.*

***Western blots:*** Equal quantities of isolated protein samples were loaded and separated using pre-cast NuPage 7-12% Tris-acetate gel (Life Technologies) and transferred at 160V for 1 hour (hr) onto a PDVF membrane (Millipore). Transferred blots were blocked in blocking solution of TBST-5% milk (Tris-buffered saline (TBS), 0.1% Tween, 5% non-fat powdered milk) for 30 minutes at room temperature (RT). Primary antibodies were diluted in blocking buffer and incubated for 1 hour at RT (Actin, Sigma A2228, 1:10000) or overnight at 4°C (FGL2) Human polyclonal FGL2 antibodies were generated in house by Dr. Gary Levy (1)^[[1]](#footnote-1)^(1:500) and alternatively the human FGL2 antibody was purchased from Abnova (6D9) (1:500). Secondary antibodies were incubated at RT in TBST with 5% skim milk, donkey anti-rabbit secondary antibody (Cedarlane, 1:10000) or anti-mouse (Abcam Ab6728, 1:10000). The membrane was developed using Clarity Western ECL Substrate (Bio-Rad) and imaged using the ChemiDoc system (Bio-Rad).

1. Foerster K, Helmy A, Zhu Y, Khattar R, Adeyi OA, Wong KM, et al. The novel immunoregulatory molecule FGL2: a potential biomarker for severity of chronic hepatitis C virus infection. J Hepatol. 2010 Oct;53(4):608–15. [↑](#footnote-ref-1)
